# Association between Dietary Mineral Intake and Chronic Kidney Disease: The Health Examinees (HEXA) Study

**DOI:** 10.3390/ijerph15061070

**Published:** 2018-05-24

**Authors:** Jeewoo Kim, Juyeon Lee, Kyoung-Nam Kim, Kook-Hwan Oh, Curie Ahn, Jongkoo Lee, Daehee Kang, Sue K. Park

**Affiliations:** 1 Department of Medicine, College of Medicine, Seoul National University, 103 Daehakro, Jongnogu, Seoul 03080, Korea; zapdos2bolt@gmail.com; 2 Department of Preventive Medicine, College of Medicine, Seoul National University, 103 Daehakro, Jongnogu, Seoul 03080, Korea; juyeon87@snu.ac.kr (J.L.); dhkang@snu.ac.kr (D.K.); 3 Department of Biomedical Sciences, College of Medicine, Seoul National University, 103 Daehakro, Jongnogu, Seoul 03080, Korea; 4 Cancer Research Institute, Seoul National University, 103 Daehakro, Jongnogu, Seoul 03080, Korea; 5 Division of Public Health and Preventive Medicine, Seoul National University Hospital, 101 Daehakro, Jongnogu, Seoul 03080, Korea; kkn002@snu.ac.kr; 6 Division of Nephrology, Department of Internal Medicine, Seoul National University Hospital, 101 Daehakro, Jongnogu, Seoul 03080, Korea; ohchris@hanmail.net (K.-H.O.); curie@snu.ac.kr (C.A.); 7 JW Lee Center for Global Medicine, College of Medicine, Seoul National University, IhwaJang-gil 71 Jongnogu, Seoul 03087, Korea; docmohw@snu.ac.kr; 8 Department of Family Medicine, Seoul National University Hospital, 101 Daehakro, Jongnogu, Seoul 03080, Korea

**Keywords:** chronic kidney disease, dietary mineral intake, Korean Genome and Epidemiologic Study

## Abstract

Few studies have explored the association between mineral intake and chronic kidney disease (CKD). A cross-sectional analysis investigated the association between mineral intake (calcium, phosphorus, sodium, potassium, iron, and zinc) and CKD using the Health Examinee (HEXA) cohort of the Korean Genome and Epidemiologic Study (KoGES). For 159,711 participants, mineral intake was assessed by a food frequency questionnaire. CKD was defined as an estimated glomerular filtration rate (eGFR) of less than 60 mL/min/1.73 m^2^. Dietary intake of each mineral was divided into quartiles and the quartile including recommended dietary allowance (RDA) or adequate intake (AI) of each mineral was used as a reference. We assessed the association between the quartile of mineral intakes and CKD using polytomous logistic regression models. The lowest quartiles of phosphorus (≤663.68 mg/day, odds ratio [OR] = 1.64, 95% confidence interval [CI]: 1.25–2.15), potassium (≤1567.53 mg/day, OR = 1.87, 95% CI: 1.27–2.75), iron (≤6.93 mg/day, OR = 1.53, 95% CI: 1.17–2.01), and zinc (≤5.86 mg/day, OR = 1.52, 95% CI: 1.02–2.26) were associated with higher odds for advanced CKD compared with the references. The present study suggests that an inadequate intake of some minerals may be associated with CKD occurrence in the general population. Due to the reverse causation issue in this cross-sectional study design, further longitudinal prospective studies are needed in order to prove the results.

## 1. Introduction

Chronic kidney disease (CKD) is defined as the existence of structural damage in the kidney or reduced kidney function (glomerular filtration rate (GFR) <60 mL/min/1.73 m^2^) lasting over three months [1]; it is one of the most important health conditions that impose substantial disease burden in the aging society [2]. The worldwide prevalence of CKD is estimated to be 8–16% [3], and it is estimated that over two million people have progressed to end stage renal disease (ESRD), which is a life-threatening outcome of CKD requiring renal replacement therapy for survival [4]. According to the Korea National Health and Nutrition Examination Survey (KNHANES) in 2011–2013, the prevalence of CKD for adults aged ≥20 in the Republic of Korea in 2013 was estimated to be 8.2% [5].

Other than established risk factors for CKD including hypertension, diabetes mellitus, poor glycemic control, and dyslipidemia [6,7], recent studies and guidelines suggested that mineral metabolism is associated with the pathogenesis or progression of CKD [8]. In 2012, the Kidney Disease Improving Global Outcomes (KDIGO) guideline emphasized the dietary impact of minerals on CKD regarding sodium, potassium, calcium, phosphorus, and even iron [7]. However, regarding specific minerals, epidemiologic evidence about the direct association between dietary mineral intake and CKD is limited, and has been conducted in small sample sizes [9]. Most studies examining the association between dietary mineral intake and CKD have focused on the terminal stages of CKD or on patients on dialysis [10,11,12], or did not distinguish the stages of CKD (early versus advanced stage of CKD) as an outcome variable [13].

Additionally, there are major differences in key food sources of nutrients such as calcium (milk), phosphorus, iron, and zinc (refined grains including white rice) between the Korean food system and the food systems of western societies [14].

In the present study, we examined the association between the dietary intake of six selected minerals (calcium, phosphorus, sodium, potassium, iron, and zinc) and the odds of early or advanced CKD using large-scale general population cohort data. We also explored the potential heterogeneity of the association between mineral intake and CKD by comorbid diseases such as hypertension and diabetes mellitus.

## 2. Materials and Methods

### 2.1. Study Population

**Figure 1:**
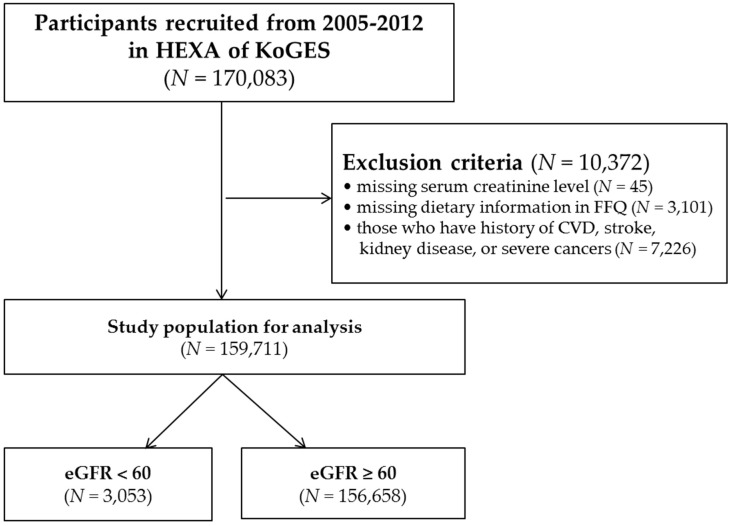
Flow chart of exclusion criteria to specify the study population.

The Health Examinees (HEXA) study is a cohort study composed of the Korean Genome and Epidemiologic Study (KoGES). Detailed information regarding the HEXA study and KoGES are presented elsewhere [15]. For a baseline, a total of 170,083 participants, aged 40 to 69 years, who visited the hospital for regular checkups from 2005 to 2012 were recruited for the HEXA cohort study of KoGES. Using the baseline data of the HEXA cohort, we conducted cross-sectional analyses in the present study. Among the participants, we excluded those without information on age or serum creatinine levels (*n* = 45), a food frequency questionnaire (FFQ) (*n* = 3101), and history of cardiovascular disease (CVD) (angina/myocardial infarction), stroke, kidney diseases (chronic kidney inflammation/renal failure), and severe cancers (liver cancer, gastric cancer, lung cancer, and colon cancer) (*n* = 7226), (Table 1). Therefore, final analysis was conducted for 159,711 participants (3053 with an estimated glomerular filtration rate (eGFR) <60 mL/min/1.73 m^2^ and 156,658 with an eGFR ≥60 mL/min/1.73 m^2^) (Figure 1). All of the subjects gave their informed consent for inclusion before they participated in the study. The protocol of this study was approved by the institutional review board (IRB) of Seoul National University Hospital (IRB number: 1607-139-778). All of the subjects gave their informed consent for inclusion before they participated in the study.

### 2.2. Definition of CKD

According to the Kidney Disease Improving Global Outcome (KDIGO) 2012 clinical practice guidelines, CKD is defined as abnormalities of kidney structure or renal function that persist for over three months. In the present study, CKD was defined as impaired kidney function determined by an eGFR <60 mL/min/1.73 m^2^ for at least three months [7]. eGFR was calculated by using the modification of diet in renal disease (MDRD) equation, and the unit was expressed in mL/min/1.73 m^2^ of body surface area. Serum creatinine levels were measured at a single time point when participants visited the hospital to receive a health checkup. The abbreviated MDRD study equation is as follows [16]:

$$\mathrm{eGFR}=\;186\;\times {\left(\mathrm{Serum}\;\mathrm{creatinine}\right)}^{-1.154}\;\times {\left(\mathrm{Age}\right)}^{-0.203}\;\times \left(0.742\;\mathrm{if}\;\mathrm{female}\right)\;\times \left(1.210\;\mathrm{if}\;\mathrm{African}\;\mathrm{American}\right),$$

CKD can be classified into five stages based on the eGFR levels of individuals. eGFR is categorized into stages 1 to 5 of CKD as follows: stage 1 (eGFR ≥90 mL/min/1.73 m^2^), stage 2 (60–89 mL/min/1.73 m^2^), stage 3A (45–59 mL/min/1.73 m^2^), stage 3B (30–44 mL/min/1.73 m^2^), stage 4 (15–29 mL/min/1.73 m^2^), and stage 5 (<15 mL/min/1.73 m^2^) [7]. To maintain a sufficient sample size in each category, we classified CKD into three groups in the present study: a non-CKD group (eGFR ≥60 mL/min/1.73 m^2^), early CKD group (45< eGFR<60 mL/min/1.73 m^2^), and advanced CKD group (<45 mL/min/1.73 m^2^).

### 2.3. Dietary Assessment

The dietary intakes of minerals were assessed using a validated semi-quantitative FFQ developed for KoGES [17]. Study participants were asked to estimate the frequencies of consumption and the average amounts of servings of 106 food items in prior years. Daily nutrient intakes were calculated by combining serving frequency per day, average amount of serving, and portion per unit for each food item.

We divided the dietary intake of minerals (calcium, phosphorus, sodium, potassium, iron, and zinc) into quartiles and used them in further analysis. The category containing the recommended dietary allowance (RDA) or adequate intake (AI) (in case RDA was unavailable) of each mineral for individuals aged 51 to 70 was designated as a reference category [18,19,20,21]; calcium–the highest quartile (Q4 ≥567.67 mg/day), phosphorus–the second-lowest quartile (Q2 = 663.69–844.27 mg/day), sodium–the second-lowest quartile (Q2 = 1541.10–2350.69 mg/day), potassium–the highest quartile (Q4 ≥2803.08 mg/day), iron–the second-lowest quartile (Q2 = 6.94–9.16 mg/day), and zinc–the third-highest quartile (Q3 = 7.38–9.35 mg/day) (Table 2).

### 2.4. Clinical and Laboratory Measurements

Height and weight were measured and body mass index (BMI) was calculated by dividing the body weight in kilograms (kg) by the height in meters (m) squared (kg/m^2^). The Blood pressure was measured with sphygmomanometers after rest, and separate values that were obtained one minute apart were averaged.

Blood and urine samples were obtained from each participant after ≥8 h of fasting. Venous blood was drawn with a Vacutainer needle (22–23 gauge) and collected in a 10-mL Serum separate (SST) tube, a 10-mL ethilen dianmin acetic acid (EDTA) tube, a 3-mL EDTA tube, and a 15-mL conical tube. For each tube, a specific two-dimensional barcode was tagged for labeling. After the sampling process and labeling, a SST tube containing at least 8 mL of a sample was kept at room temperature for 20–30 min, then centrifuged at 2500 rpm for 10 min, and finally kept refrigerated at 4 °C. EDTA tubes were put on a roller mixer for 5 min, to ensure that the samples were correctly mixed with the anti-coagulant, and they were then kept refrigerated. These samples were analyzed in the central laboratory in KoGES, of which the reliability of biomarker analysis was published [22]. Serum creatinine levels were determined by Jaffe assay using a Hitachi Automatic Analyzer 7180 (Hitachi, Tokyo, Japan) from 2004 to 2008, and an ADVIA 1650 Auto Analyzer (Siemens, Plano, TX, USA) in 2008, by a compensated rate blanked Jaffe kinetic assay using a Hitachi Automatic Analyzer 7180 (Hitachi, Tokyo, Japan) from 2008 to 2009, a Hitachi Automatic Analyzer 7600 (Hitachi, Japan) from 2009 to 2011, and a Modular Analytic (Hitachi, Tokyo, Japan) from 2012 to 2013 [15].

### 2.5. Statistical Analysis

To compare the socio-demographic characteristics of the CKD (eGFR <60 mL/min/1.73 m^2^) and non-CKD (eGFR ≥60 mL/min/1.73 m^2^) groups, we performed the Pearson chi-square test for categorical variables and the independent *t*-test for continuous variables. We evaluated the associations between dietary mineral intake and early and advanced CKD using polytomous logistic regression models.

We also performed stratified analysis by hypertension and diabetes mellitus status by using the same logistic regression models. Hypertension was defined as a person with anti-hypertensive medication or systolic BP ≥ 140 mmHg, diastolic BP ≥ 90 mmHg, or the presence of a medical history of hypertension. Diabetes mellitus was defined as fasting blood glucose ≥ 126 mg/mL or the presence of a history of diabetes.

**Figure 2:**
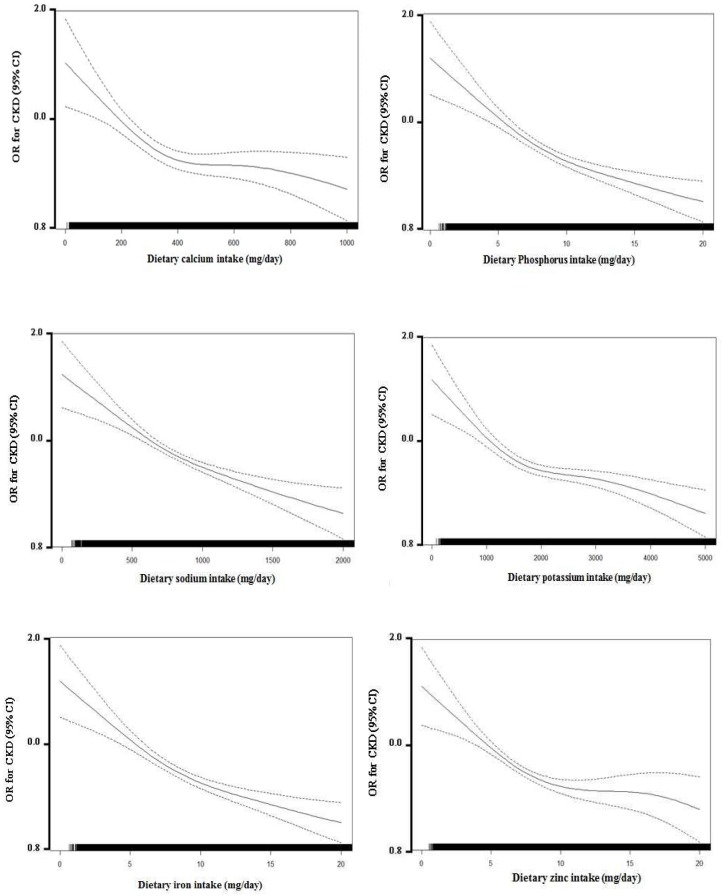
Multivariate association of continuously measured dietary mineral intake levels (mg/day) and chronic kidney disease (CKD).

We selected covariates by backward elimination methods (stay significance levels: 0.05; the first covariates set: age, sex, monthly household income, education levels, drinking habits, passive smoking, weight, energy intake per day, BMI, regular exercise, history of smoking, hypertension, diabetes, serum albumin, protein intake per day, uric acid, and total cholesterol levels). Final models included the following covariates: age (year), sex (man/woman), energy intake per day (<1697.33 kcal/day/≥1697.33 kcal/day – median), BMI (<23.71 kg/m^2^/≥23.71kg/m^2^ – median), and regular exercise (no/yes), histories of smoking (no/yes, including ex-smokers and current smokers), hypertension (no/yes), diabetes (no/yes), serum albumin levels (<4.6 g/dL/≥4.6 g/dL – median), protein intake per day (<55.27g/day/≥55.27g/day – median), uric acid levels (<4.5 mg/dL/≥4.5 mg/dL – median), and total cholesterol levels (<196 mg/dL/≥196 mg/dL – median). We examined the multicollinearity between independent variables with the Pearson correlation coefficient and variance inflation factor. The spline model was adjusted for age, sex, and confounding factors (Figure 2). All of the analyses were conducted using SAS version 9.3 (SAS Institute Inc., Cary, NC, USA) and a *p*-value < 0.05 was considered statistically significant.

## 3. Results

### 3.1. Baseline Characteristics

Of the 159,711 participants, 3053 (1.9%) were classified as having CKD. Table 1 shows the baseline characteristics of the CKD and non-CKD groups. Compared with the participants without CKD, those with CKD were more likely to be older, male, smokers who exercised more, were single (unmarried), had lower education levels (below middle school), had a monthly household income lower than 1,500,000 KRW, lower protein and total cholesterol levels, and a history of hypertension and diabetes mellitus (Table 1). Distributions of the dietary intakes of selected minerals (calcium, phosphorus, sodium, potassium, iron, and zinc) according to CKD status are shown in Table S1 and Figure S1.

### 3.2. The Associations between Dietary Mineral Intake and CKD

The associations between dietary mineral intake and early or advanced CKD are demonstrated in Table 2. When we divided the mineral intake into quartiles, the lowest quartiles of mineral intake were associated with increased odds of advanced CKD compared with the reference quartiles, which included values for the recommended dietary allowance (RDA) or adequate intake (AI), for phosphorus (odds ratio [OR] = 1.64; 95% confidence interval [CI]: 1.25–2.15), potassium (OR = 1.87; 95% CI: 1.27–2.75), iron (OR = 1.53; 95% CI: 1.17–2.01), and zinc (OR = 1.52; 95% CI: 1.02–2.26). As shown in Tables S3 and S4, when we conducted analyses using the Chronic Kidney Disease Epidemiology Collaboration (CKD-EPI) and Asian-modified CKD-EPI (aCKD-EPI) equations, the results were robust and similar to the results of the MDRD equation. Additionally, mineral intake levels differed according to protein intake levels (Table S5).

### 3.3. The Associations between Dietary Mineral Intake and CKD Stratified by Hypertension Status

When we stratified the analysis by hypertensive and non-hypertensive groups, the associations between mineral intake and CKD were only observed in the hypertensive groups (Table 3). This result also showed that 5126 subjects had diabetes mellitus among the hypertensive patients. However, when we stratified the analysis by diabetes mellitus status, the associations between mineral intake and CKD were observed in both diabetes mellitus and non-diabetes mellitus groups, and the pattern of the associations were similar (Table S2).

## 4. Discussion

In the present study conducted among the general population, we found that dietary intakes of phosphorus, potassium, iron, and zinc that were lower than the recommended values were associated with higher odds of advanced CKD.

A previous cross-sectional study conducted in Australia reported a dose-dependent pattern in the associations between calcium and phosphorus intake and CKD [13]. Another cohort study conducted in Iran demonstrated inverse associations between potassium and magnesium intake and incident CKD among adults [9]. In the present study, we found inverse associations between intakes of phosphorus, potassium, iron, and zinc and advanced CKD, while we did not find any association between calcium intake and CKD, which might be due to differences in ethnicity, the size of the population, methods of assessing of dietary intake, and adjusted covariates.

Previous studies suggested the deleterious effects of hyperphosphatemia on promoting death and kidney failure in CKD patients, and emphasized the dietary restrictions of phosphorus or phosphate binders as means of managing hyperphosphatemia [23,24,25]. However, in a prior study conducted among the general population, those with higher phosphorus intake (quintile 5 in the general population), relative to the population with phosphorous intake at the lowest quintile, was inversely associated with the likelihood of CKD [13], which is consistent with our result. These discrepancies can be attributed to a variety of reasons, including differences in the characteristics of the study population, outcome variables, and clinical setting. The biggest reason between the former studies [23,24,25] and the latter studies [13], including our study, is that the former studies [23,24,25] have shown that phosphorous intake is associated with the risk of ESRD exacerbations and death in most of the patients with stage 3 or higher CKD; whereas the latter studies have shown that phosphorous intake is associated with the likelihood of CKD morbidity in the general population, where CKD patients with three or more stages are less common (about 25% [mean age = 65 years] or 15% [mean age = 66 years] in two Italian studies [13] and only 1.8% in the Korean population [this study]). Due to the complex interaction between FGF23 [26], phosphorus metabolism [9], and CKD progression [27], further studies exploring the relationship between dietary phosphorus and CKD are needed.

To our knowledge, there have been no randomized controlled trials assessing the association between dietary potassium on the prevalence of CKD. However, it has been suggested that high potassium intake may be protective against CKD by its physiologic functions, including lowering blood pressure (BP) or acting as a vascular protectant [28]. Previous studies have recommended that patients with advanced CKD should meet a daily intake of potassium of up to 4700 mg/day, which belongs to the reference category of potassium intake (Q4 ≥2803.08 mg/day) in the present study [29].

There have been few studies directly investigating the relationship between dietary iron intake and CKD [30]. Previous studies have suggested that anemia may aggravate CKD, and pointed out that the early prevention of anemia is important in CKD patients [31,32,33]. Since oral iron supplementation is effective in preventing the progression of anemia [34], sufficient iron intake may prevent the development or progression of CKD by correcting anemia.

Similar to iron, there have been limited studies directly investigating the association between dietary zinc intake and CKD. Zinc deficiency can induce arterial stiffness or a loss of flexibility in the vascular wall, resulting in elevated blood pressure and an increased likelihood of CVD, and possibly CKD as well, especially among hypertensive individuals [35,36]. Further studies are warranted to confirm the present results and suggested mechanisms.

We found the heterogeneity of the association between dietary mineral intake and CKD by hypertension status. Hypertension is the second leading cause of ESRD, and contributes to the progression of CKD [37]. Since hypertension can induce an increased likelihood of CKD, individuals with hypertension may be more susceptible to mineral deficiency with regards to CKD than those without hypertension. How hypertension affects the progression of CKD is explained by the transmission of high systemic BP to the renal vascular bed, and renal injury via BP-independent of angiotensin II [37]. Detailed discussions are presented in the Supplementary Material.

Due to the present study’s cross-sectional design, the possibility of reverse causality cannot be ruled out. As CKD progresses toward ESRD, CKD patients are more likely to experience anorexia, resulting in poor nutrient intake and uremic malnutrition [38]. However, in the stratified analysis, the associations between mineral intake and CKD differed by hypertension status, and point estimates for the associations between higher iron and zinc intake and advanced CKD were below 1. These results cannot be explained by uremic malnutrition, and suggest that reverse causality alone is not responsible for the observed associations. Nevertheless, it is observed that low mineral intake is related to early CKD, as well as advanced CKD, so not all of the results are caused by reverse causation due to food restriction. Koreans have lower calcium, potassium, ion, and zinc intake than Westerners, while intake of phosphorus is similar, and sodium intake is higher. It is necessary to correct the nutritional imbalance of the Korea food context [14]. Further studies, especially with longitudinal study design, are warranted in order to confirm the results. There are several limitations to consider. First, owing to the cross-sectional design of the study, we cannot establish the causality between mineral intake and CKD. Second, the study participants were recruited in several regions of the urban area, and may not represent the population of the Republic of Korea. Third, although we used a validated semi-quantitative food frequency questionnaire (FFQ), it did not cover all kinds of food sources. Also, the bioavailability of each form of food source was not considered in the FFQ. Fourth, dietary plasma/urine biomarkers for nutrients were not measured in this study. However, the validation and reproductive tests of the FFQ used in the study were conducted in a previous KoGES study [17]. Fifth, since our calcium intake level is much lower than that of Europe and the United States, the results of this study are difficult to apply to the other population, especially with higher calcium intakes [14]. Sixth, sodium intake was estimated by using the FFQ, which are prone to underestimate of sodium intake [39]. Also, it is difficult to measure the uptake of mixed meals and salt in sauces. Finally, the definition of CKD was solely based on serum creatinine measurements, which were not repeated for three months. The MDRD equation is widely used in clinical practice in Korea [40,41]. Also, the smallest bias and highest accuracy were observed for the MDRD equation among MDRD, CKD-EPI, Cockcroft–Gault equations [42], although ethnic heterogeneity can be present. In the present study, the association between dietary mineral intake and CKD remained consistent regardless of the equations (Tables S3 and S4).

However, the present study has some strengths. The large sample size of this study provides sufficient statistical power. In addition, the present study assesses various minerals and stages of CKD, providing novel evidence to the issues that were not investigated sufficiently.

In conclusion, lower mineral intake than recommended values was associated with an increased likelihood of advanced stages of CKD. The present study suggests that the adequate dietary intake of minerals close to or above the recommended values is important for maintaining kidney function in the general population.

## 5. Conclusions

Deficient intake of these minerals (phosphorus, potassium, iron, and zinc) was associated with an increased likelihood of advanced CKD. Otherwise, the intrinsic traits of subjects with advanced CKD other than nutritional intake, such as uremic malnutrition, may have resulted in the lower intake of the minerals.

## Figures and Tables

**Table 1:** General characteristics of the study population stratified by chronic kidney disease (CKD)^1^ status in the Health Examinees Study (HEXA) of a major urban cohort in the Korea Genome and Epidemiologic Study (KoGES), 2005–2012.

	CKD ^1^ *(N* = 3053)	Non-CKD ^1^ (*N* = 156,658)	*p*-Value
	*N* (%)	*N* (%)	
Sex			
Male	1200 (39.3)	52,680 (33.6)	<0.01
Female	1843 (60.7)	103,978 (66.4)	
Marital status			
Single	466 (15.3)	16,624 (10.6)	<0.01
Married	2426 (79.5)	131,889 (84.2)	
Others	161 (5.3)	8145 (5.2)	
Education			
Below middle school	905 (29.6)	26,265 (16.8)	<0.01
High school	1387 (45.4)	86,169 (55.0)	
Higher than college	699 (22.9)	41,926 (26.8)	
Monthly household income (KRW)			
<1,500,000	554 (18.2)	14,705 (9.4)	<0.01
1,500,000–2,999,999	579 (19.0)	26,275 (16.8)	
3,000,000–3,999,999	836 (27.4)	56,592 (36.1)	
≥4,000,000	378 (12.4)	33,472 (21.4)	
Regular exercise ^2^			
No	1382 (45.3)	74,342 (47.5)	0.06
Yes	1660 (54.4)	81,751 (52.2)	
Drinking			
No	1788 (58.6)	78,378 (50.0)	<0.01
Yes	1249 (40.9)	77,633 (49.6)	
Smoking			
No	1566 (51.3)	61,049 (39.0)	<0.01
Yes	537 (17.6)	20,572 (13.1)	
Passive smoking ^3^			
No	2230 (73.0)	107,865 (68.9)	<0.01
Yes	658 (21.6)	40,542 (25.9)	
Hypertension ^4^			
No	1234 (40.4)	113,189 (72.2)	<0.01
Yes	1819 (59.6)	43,469 (27.8)	
Diabetes ^5^			
No	2448 (80.2)	147,458 (94.1)	<0.01
Yes	605 (19.8)	9200 (5.9)	
Use of dietary supplements			
No	2607 (85.4)	139,902 (89.3)	<0.01
Yes	413 (13.5)	15,636 (10.0)	
	**Mean (SD)**	**Mean (SD)**	
Age (year)	60.58 (7.84)	52.41 (8.28)	<0.01
Height (cm)	159.8 (8.48)	160.5 (8.02)	<0.01
Weight (kg)	63.63 (10.32)	61.77 (9.92)	<0.01
Body Mass Index (BMI) (kg/m^2^)	24.83 (3.02)	23.90 (2.90)	<0.01
Albumin (g/dL)	4.60 (0.33)	4.64 (0.27)	<0.01
Protein intake (g)	56.02 (24.04)	60.36 (27.52)	<0.01
Creatinine (mg/dL)	1.40 (0.96)	0.81 (0.16)	<0.01
Uric acid (mg/dL)	6.11 (1.76)	4.68 (1.26)	<0.01
Total cholesterol (mg/dL)	199.6 (39.41)	197.9 (35.22)	0.02

^1^ Chronic kidney disease (CKD) was defined as an eGFR (estimated glomerular filtration rate) of less than 60 mL/min/1.73 m^2^ on the basis of the National Kidney Foundation’s Kidney Disease Outcome Quality Initiative working group definition [7]; ^2^ Regular exercise was defined as performing regular exercise enough to sweat once a week or more; ^3^ Passive smoking (among subjects who had never smoked) was determined by asking. “How many times do you indirectly inhale smoke from other people at home or your workplace?” ^4^ Hypertension was defined as a person with anti-hypertensive medication or systolic blood pressure ≥140, diastolic blood pressure ≥90 mm/Hg, or the presence of history of hypertension; ^5^ Diabetes was defined as fasting blood glucose ≥126 mg/mL, or the presence of a history of diabetes.

**Table 2:** Association between dietary mineral intake and CKD in the Health Examinees (HEXA) study of the Korea Genome and Epidemiologic Study (KoGES), 2005–2012.

Mineral Intake	Non-CKD ^1^ (*n*= 156,658)	Early stage CKD ^2^ (*n* = 2573)		Advanced stage CKD ^2^ (*n* = 480)	
	*n* (%)	*n* (%)	OR (95% CI) ^3^	*n* (%)	OR (95% CI) ^3^
Calcium (mg/day)					
Q1 (<273.37)	39,057 (24.9)	715 (27.8)	0.93 (0.79–1.08)	155 (32.3)	1.27 (0.88–1.82)
Q2 (273.38–401.36)	39,169 (25.0)	622 (24.2)	0.94 (0.82–1.08)	137 (28.5)	1.33 (0.96–1.85)
Q3 (401.37–567.66)	39,206 (25.0)	623 (24.2)	0.95 (0.83–1.08)	99 (20.6)	1.05 (0.76–1.45)
Q4 (≥567.67)	39,226 (25.0)	613 (23.8)	Reference	89 (18.5)	Reference
Phosphorus (mg/day)					
Q1 (<663.68)	39,004 (24.9)	739 (28.7)	1.04 (0.92–1.17)	184 (38.3)	1.64 (1.25–2.15)
Q2 (663.69–844.27)	39,173 (25.0)	646 (25.1)	Reference	109 (22.7)	Reference
Q3 (844.28–1067.44)	39,186 (25.0)	637 (24.8)	1.12 (0.95–1.32)	105 (21.9)	1.07 (0.72–1.59)
Q4 (≥1067.45)	39,295 (25.1)	551 (21.4)	1.06 (0.87–1.29)	82 (17.1)	0.87 (0.54–1.38)
Sodium (mg/day)					
Q1 (<1541.09)	39,089 (25.0)	696 (27.1)	1.02 (0.90–1.15)	142 (29.6)	0.96 (0.74–1.24)
Q2 (1541.10–2350.69)	39,163 (25.0)	630 (24.5)	Reference	135 (28.1)	Reference
Q3 (2350.70–3260.41)	39,218 (25.0)	595 (23.1)	0.93 (0.82–1.05)	115 (24.0)	0.94 (0.71–1.22)
Q4 (≥3260.42)	39,188 (25.0)	652 (25.3)	1.02 (0.89–1.15)	88 (18.3)	0.74 (0.54–1.00)
Potassium (mg/day)					
Q1 (<1567.53)	39,015 (24.9)	731 (28.4)	1.02 (0.86–1.20)	181 (37.7)	1.86 (1.27–2.74)
Q2 (1567.54–2114.26)	39,166 (25.0)	650 (25.3)	1.01 (0.87–1.17)	112(23.3)	1.27 (0.89–1.83)
Q3 (2114.27–2803.07)	39,204 (25.0)	618 (24.0)	1.03 (0.90–1.17)	106 (22.1)	1.30 (0.94–1.79)
Q4 (≥2803.08)	39,273 (25.1)	574 (22.3)	Reference	81 (16.9)	Reference
Iron (mg/day)					
Q1 (<6.93)	38,992 (24.9)	753 (29.3)	1.04 (0.92–1.17)	182 (37.9)	1.53 (1.17–2.01)
Q2 (6.94–9.16)	39,160 (25.0)	659 (25.6)	Reference	109 (22.7)	Reference
Q3 (9.17–12.12)	39,202 (25.0)	618 (24.0)	1.02 (0.89–1.17)	108 (22.5)	1.08 (0.78–1.49)
Q4 (≥12.13)	39,304 (25.1)	543 (21.1)	0.93 (0.79–1.09)	81 (16.9)	0.79 (0.54–1.16)
Zinc (mg/day)					
Q1 (<5.86)	39,019 (24.9)	729 (28.3)	1.00 (0.84–1.19)	178 (37.1)	1.52 (1.02–2.25)
Q2 (5.87–7.37)	39,136 (25.0)	681 (26.5)	1.02 (0.88–1.18)	112 (23.3)	1.06 (0.74–1.51)
Q3 (7.38–9.35)	39,227 (25.0)	604 (23.5)	Reference	97 (20.2)	Reference
Q4 (≥9.36)	39,276 (25.1)	559 (21.7)	1.00 (0.88–1.14)	93 (19.4)	0.96 (0.70–1.32)

^1^ Chronic kidney disease (CKD) was defined as the eGFR (estimated GFR) of less than 60 mL/min/1.73 m^2^ on the basis of the National Kidney Foundation’s Kidney Disease Outcome Quality Initiative working group definition [7]; ^2^ Early stage CKD was defined as 45 ≤ eGFR < 60 mL/min/1.73 m^2^, and advanced stage CKD was defined as eGFR (estimated GFR) of less than 45 mL/min/1.73 m^2^; ^3^ Adjusted for age, sex, energy intake per day, body mass index, regular exercise, smoking status, history of hypertension and diabetes, albumin, protein intake per day, use of dietary supplements, uric acid, and cholesterol.

**Table 3:** Association between dietary mineral intake and CKD stages stratified by hypertension status in the Health Examinees (HEXA) study of the Korea Genome and Epidemiologic Study (KoGES), 2005–2012.

Mineral Intake	Hypertension ^4^ (*N* = 45,288)					Non-Hypertension ^4^ (*N* = 114,423)				
	Non-CKD ^1^ (*N* = 43,469)	Early Stage CKD ^2^ (N = 1457)		Advanced Stage CKD ^2^ (*N* = 362)		Non-CKD ^1^ (*N* = 113,189)	Early Stage CKD ^2^ (*N* = 1116)		Advanced Stage CKD ^2^ (*N* = 118)	
	*N* (%)	N (%)	OR (95% CI) ^3^	N (%)	OR (95% CI) ^3^	*N* (%)	*N* (%)	OR (95% CI) ^3^	*N* (%)	OR (95% CI) ^3^
Phosphorus (mg/day)										
Q1 (<663.68)	11,299 (26.0)	432 (29.6)	1.08 (0.92–1.25)	152 (42.0)	1.79 (1.34–2.39)	27,705 (24.5)	307 (27.5)	0.94 (0.80–1.12)	32 (27.1)	1.03 (0.60–1.74)
Q2 (663.69–844.27)	11,099 (25.5)	358 (24.6)	Reference	81 (22.4)	Reference	28,074 (24.8)	288 (25.8)	Reference	28 (23.7)	Reference
Q3 (844.28–1067.44)	10,754 (24.7)	348 (23.9)	1.11 (0.90–1.36)	71 (19.6)	0.96 (0.62–1.49)	28,432 (25.1)	289 (25.9)	1.20 (0.96–1.50)	34 (28.8)	1.10 (0.56–2.19)
Q4 (≥1067.45)	10,317 (23.7)	319 (21.9)	1.14 (0.89–1.46)	58 (16.0)	0.82 (0.49–1.37)	28,978 (25.6)	232 (20.8)	1.07 (0.81–1.41)	24 (20.3)	0.84 (0.38–1.89)
*P*-interaction			0.45		<0.01					
Potassium (mg/day)										
Q1 (<1567.53)	11,506 (26.5)	427 (29.3)	0.97 (0.79–1.20)	148 (40.9)	1.83 (1.21–2.78)	27,509 (24.3)	304 (27.2)	0.97 (0.77–1.23)	33 (28.0)	1.28 (0.63–2.62)
Q2 (1567.54–2114.26)	10,957 (25.2)	361 (24.8)	0.95 (0.79–1.15)	84 (23.2)	1.18 (0.80–1.76)	28,209 (24.9)	289 (25.9)	1.05 (0.85–1.29)	28 (23.7)	1.16 (0.62–2.19)
Q3 (2114.27–2803.07)	10,542 (24.2)	340 (23.3)	0.97 (0.83–1.14)	73 (20.2)	1.17 (0.82–1.68)	28,662 (25.3)	278 (24.9)	1.09 (0.91–1.31)	33 (28.0)	1.35 (0.78–2.32)
Q4 (≥2803.08)	10,464 (24.1)	329 (22.6)	Reference	57 (15.8)	Reference	28,809 (25.4)	245 (21.9)	Reference	24 (20.3)	Reference
*P*-interaction			0.61		<0.01					
Iron (mg/day)										
Q1 (<6.93)	11,359 (26.1)	429 (29.4)	1.04 (0.89–1.21)	152 (42.0)	1.71 (1.28–2.28)	27,633 (24.4)	324 (29.0)	1.02 (0.86–1.21)	30 (25.4)	1.01 (0.59–1.75)
Q2 (6.94–9.16)	10,947 (25.2)	367 (25.2)	Reference	83 (22.9)	Reference	28,213 (24.9)	292 (26.2)	Reference	26 (22.0)	Reference
Q3 (9.17–12.12)	10,797 (24.8)	340 (23.3)	1.01 (0.85–1.20)	69 (19.1)	0.91 (0.63–1.31)	28,405 (25.1)	278 (24.9)	1.02 (0.84–1.23)	39 (33.0)	1.62 (0.92–2.84)
Q4 (≥12.13)	10,366 (23.9)	321 (22.0)	1.02 (0.84–1.25)	58 (16.0)	0.80 (0.53–1.23)	28,938 (25.6)	222 (19.9)	0.84 (0.67–1.05)	23 (19.5)	1.02 (0.51–2.02)
*P*-interaction			0.53		<0.01					
Zinc (mg/day)										
Q1 (<5.86)	11,216 (25.8)	432 (29.6)	1.16 (0.93–1.43)	146 (40.3)	1.85 (1.19–2.88)	27,804 (24.6)	297 (26.6)	0.82 (0.65–1.03)	32 (27.1)	0.82 (0.40–1.68)
Q2 (5.87–7.37)	10,993 (25.3)	379 (26.0)	1.11 (0.92–1.34)	86 (23.8)	1.20 (0.80–1.79)	28,142 (24.9)	302 (27.1)	0.93 (0.76–1.14)	26 (22.0)	0.77 (0.40–1.47)
Q3 (7.38–9.35)	10,855 (25.0)	334 (22.9)	Reference	68 (18.8)	Reference	28,372 (25.1)	270 (24.2)	Reference	29 (24.6)	Reference
Q4 (≥9.36)	10,405 (23.9)	312 (21.4)	1.03 (0.87–1.21)	62 (17.1)	0.95 (0.67–1.37)	28,871 (25.5)	247 (22.1)	1.01 (0.84–1.21)	31 (26.3)	1.22 (0.71–2.07)
*P*-interaction			0.59		<0.01					

^1^ Chronic kidney disease (CKD) was defined as the eGFR (estimated GFR) of less than 60 mL/min/1.73 m^2^ on the basis of the National Kidney Foundation’s Kidney Disease Outcome Quality Initiative working group definition [7]; ^2^ Early stage CKD was defined as 45≤ eGFR <60 mL/min/1.73 m^2^, and advanced stage CKD was defined as an eGFR (estimated GFR) of less than 45 mL/min/1.73 m^2^; ^3^ Adjusted for age, sex, energy intake per day, body mass index, regular exercise, smoking status, history of hypertension and diabetes, albumin, protein intake per day, the use of dietary supplements, uric acid, and cholesterol; ^4^ Hypertension was defined as a person with anti-hypertensive medication or systolic blood pressure ≥140, diastolic blood pressure ≥90 mm/Hg, or the presence of a history of hypertension.

## Supplementary Materials

The following are available online at http://www.mdpi.com/1660-4601/15/6/1070/s1, Table S1: Geometric mean and inter-quartile range of dietary mineral intake according to CKD stages in the Health Examinees (HEXA) Study of the Korea Genome and Epidemiologic Study (KoGES), 2005–2012, Table S2: Association between dietary mineral intake and CKD stages stratified by diabetes status in the Health Examinees (HEXA) Study of the Korea Genome and Epidemiologic Study (KoGES), 2005–2012, Table S3: Association between dietary mineral intake and CKD (using the CKD-EPI equation) in the Health Examinees (HEXA) study of the Korea Genome and Epidemiologic Study (KoGES), 2005–2012, Table S4: Association between dietary mineral intake and CKD (using the Asian modified CKD-EPI equation) in the Health Examinees (HEXA) study of the Korea Genome and Epidemiologic Study (KoGES), 2005–2012, Table S5: Association between dietary mineral intake and protein intake status in the Health Examinees (HEXA) study of the Korea Genome and Epidemiologic Study (KoGES), 2005–2012, Tables S6~S8: General characteristics of study population stratified by each dietary mineral intake status in the Health Examinees (HEXA) study of the Korea Genome and Epidemiologic Study (KoGES), 2005–2012.
