# Supplementary material for: Association between Dietary Mineral Intake and Chronic Kidney Disease: The Health Examinees (HEXA) Study
Source: Int J Environ Res Public Health. 2018 May 24;15(6):1070. doi: 10.3390/ijerph15061070 (PMC6025644; doi:10.3390/ijerph15061070)
Supplement: Supplementary file 1 [file ijerph-15-01070-s001.pdf]

**Table S1.** Geometric mean and inter-quartile range of dietary mineral intake according to CKD stages in the Health Examinees (HEXA) Study of the Korea Genome and Epidemiologic Study (KoGES), 2005-2012

| | Non-CKD[1] | | CKD[1] | | | |
|---|---|---|---|---|---|---|
| | | | Early stage | Advanced stage | | |
| **Diet intake** | **eGFR≥90** (N=77,494) | **60≤eGFR<90** (N=79,164) | **45≤eGFR<60** (N=2,573) | **30≤eGFR<45** (N=310) | **eGFR<30** (N=170) | p-value[2] |
| | Geometric mean (IQR) | Geometric mean (IQR) | Geometric mean (IQR) | Geometric mean (IQR) | Geometric mean (IQR) | |
| **Calcium** | | | | | | |
| Total | 417.11 (325.43-554.16) | 418.60 (327.10-555.55) | 431.39 (335.22-567.43) | 414.44 (336.85-541.72) | 409.02 (330.69-534.98) | **<0.01** |
| Men | 360.88 (285.18-483.63) | 367.72 (289.46-490.75) | 385.40 (299.03-500.00) | 381.54 (304.10-477.26) | 351.96 (269.02-468.65) | **<0.01** |
| Women | 443.89 (348.91-580.38) | 451.83 (356.51-587.89) | 463.17 (364.07-594.91) | 442.46 (350.42-551.47) | 450.35 (364.89-560.13) | **<0.01** |
| p-value[3] | **<0.01** | **<0.01** | **<0.01** | **<0.01** | **<0.01** | |
| **Phosphorus** | | | | | | |
| Total | 890.81 (793.14-999.62) | 890.19 (794.30-998.30) | 895.31 (801.08-1005.50) | 883.09 (790.59-993.91) | 878.02 (793.79-980.94) | **<0.01** |
| Men | 855.39 (766.24-956.47) | 858.96 (770.18-961.87) | 866.61 (778.52-966.65) | 864.98 (778.50-976.79) | 838.01 (757.54-951.44) | **<0.01** |
| Women | 906.51 (806.71-1015.78) | 909.13 (811.89-1017.83) | 913.89 (819.45-1026.66) | 897.79 (798.57-1005.84) | 905.07 (817.99-983.69) | **<0.01** |
| p-value[3] | **<0.01** | **<0.01** | **<0.01** | 0.07 | **<0.01** | |
| **Sodium** | | | | | | |
| Total | 2296.97 (1744.46-3141.42) | 2321.24 (1759.76-3183.01) | 2390.49 (1804.84-3290.95) | 2247.19 (1701.72-2999.08) | 2291.23 (1725.55-3047.59) | **<0.01** |
| Men | 2291.10 (1730.49-3180.20) | 2302.63 (1730.76-3200.04) | 2300.72 (1703.47-3212.88) | 2368.13 (1906.42-3097.73) | 2129.94 (1576.80-2941.33) | **<0.01** |
| Women | 2299.50 (1749.50-3124.78) | 2332.27 (1775.69-3172.22) | 2448.79 (1860.44-3343.06) | 2154.63 (1631.74-2860.36) | 2400.94 (1786.71-3218.20) | **<0.01** |
| p-value[3] | **<0.01** | **<0.01** | **<0.01** | **0.02** | 0.10 | |
| **Potassium** | | | | | | |
| Total | 2193.44 (1826.01-2691.54) | 2208.29 (1838.40-2706.13) | 2217.79 (1839.42-2729.70) | 2122.78 (1756.72-2577.62) | 2148.77 (1864.78-2599.38) | **<0.01** |
| Men | 2024.57 (1689.60-2483.69) | 2063.54 (1720.28-2533.66) | 2091.05 (1752.71-2549.18) | 2059.14 (1756.27-2490.33) | 1989.48 (1619.85-2399.91) | **<0.01** |
| Women | 2270.41 (1890.81-2770.06) | 2298.40 (1918.07-2793.76) | 2301.62 (1907.57-2812.36) | 2174.87 (1779.65-2603.86) | 2257.48 (1935.45-2749.37) | **<0.01** |
| p-value[3] | **<0.01** | **<0.01** | **<0.01** | 0.04 | **<0.01** | |
| **Iron** | | | | | | |
| Total | 9.81 (8.29-11.61) | 9.79 (8.29-11.57) | 9.83 (8.42-11.61) | 9.63 (8.20-11.44) | 9.54 (8.38-11.12) | **<0.01** |
| Men | 9.08 (7.73-10.75) | 9.21 (7.83-10.88) | 9.39 (8.03-10.96) | 9.24 (7.84-10.91) | 9.05 (7.86-10.66) | **<0.01** |
| Women | 10.14 (8.57-11.90) | 10.15 (8.60-11.93) | 10.12 (8.66-11.93) | 9.95 (8.31-11.75) | 9.87 (8.87-11.30) | **<0.01** |
| p-value[3] | **<0.01** | **<0.01** | **<0.01** | 0.06 | **0.02** | |
| **Zinc** | | | | | | |
| Total | 7.87 (7.09-8.66) | 7.86 (7.10-8.66) | 7.96 (7.21-8.77) | 8.03 (7.26-8.77) | 7.91 (7.22-8.70) | **<0.01** |
| Men | 7.73 (6.90-8.57) | 7.73 (6.93-8.57) | 7.84 (7.05-8.76) | 7.76 (6.85-8.67) | 7.78 (7.13-8.83) | **<0.01** |
| Women | 7.93 (7.16-8.69) | 7.94 (7.19-8.71) | 8.04 (7.32-8.78) | 8.25 (7.45-8.82) | 8.00 (7.40-8.59) | **<0.01** |
| p-value[3] | **<0.01** | **<0.01** | **<0.01** | 0.09 | 0.09 | |

[1] Chronic kidney disease (CKD) was defined as the eGFR (estimated GFR) of less than 60 mL/min/1.73 m2 on the basis of the National Kidney Foundation's Kidney Disease Outcome Quality Initiative working group definition (K/DOQI clinical practice guidelines for chronic kidney disease: evaluation, classification, and stratification. Am J Kidney Dis 2002;39(suppl 1):S1–266.)

[2] Age, sex and total energy intake (kcal) adjusted.

[3] Age and total energy intake (kcal) adjusted.

**Table S2.** Association between dietary mineral intake and CKD stages stratified by diabetes status in the Health Examinees (HEXA) study of the Korea Genome and Epidemiologic Study (KoGES), 2005-2012

| | **Diabetes mellitus (N=9,805)** | | | | | **Non-Diabetes mellitus (N=149,906)** | | | | |
|---|---|---|---|---|---|---|---|---|---|---|
| | **Non-CKD[1]** | **Early stage CKD[2]** | | **Advanced stage CKD[2]** | | **Non-CKD[1]** | **Early stage CKD[2]** | | **Advanced stage CKD[2]** | |
| **Mineral intake** | **(N=9,200)** | **(N=444)** | | **(N=161)** | | **(N=147,458)** | **(N=2,129)** | | **(N=319)** | |
| | N (%) | N (%) | OR (95% CI)[3] | N (%) | OR (95% CI)[3] | N (%) | N (%) | OR (95% CI)[3] | N (%) | OR (95% CI)[3] |
| **Phosphorus (mg/day)** | | | | | | | | | | |
| Q1 (< 663.68) | 2,549 (27.7) | 135 (30.4) | 0.86 (0.66-1.12) | 71 (44.1) | **1.61 (1.05-2.48)** | 36,455 (24.7) | 604 (28.4) | 1.05 (0.92-1.19) | 113 (35.4) | **1.50 (1.10-2.05)** |
| Q2 (663.69-844.27) | 2,331 (25.3) | 121 (27.2) | Reference | 37 (23.0) | Reference | 36,842 (25.0) | 525 (24.7) | Reference | 72 (22.6) | Reference |
| Q3 (844.28-1067.44) | 2,207 (24.0) | 84 (18.9) | 0.93 (0.63-1.38) | 27 (16.8) | 0.83 (0.41-1.64) | 36,979 (25.1) | 553 (26.0) | **1.18 (1.00-1.40)** | 78 (24.5) | 1.08 (0.70-1.68) |
| Q4 (≥ 1067.45) | 2,113 (23.0) | 104 (23.4) | 1.56 (0.98-2.48) | 26 (16.2) | 0.91 (0.41-2.01) | 37,182 (25.2) | 447 (21.0) | 1.04 (0.85-1.28) | 56 (17.6) | 0.82 (0.48-1.38) |
| P-interaction | | | **< 0.01** | | **< 0.01** | | | | | |
| **Potassium (mg/day)** | | | | | | | | | | |
| Q1 (< 1567.53) | 2,672 (29.0) | 143 (32.2) | 0.96 (0.66-1.40) | 67 (41.6) | 1.32 (0.71-2.47) | 36,343 (24.7) | 588 (27.6) | 0.97 (0.82-1.15) | 114 (35.7) | **1.84 (1.18-2.85)** |
| Q2 (1567.54-2114.26) | 2,294 (24.9) | 118 (26.6) | 1.04 (0.74-1.47) | 39 (24.2) | 0.99 (0.55-1.80) | 36,872 (25.0) | 532 (25.0) | 0.99 (0.85-1.15) | 73 (22.9) | 1.26 (0.84-1.89) |
| Q3 (2114.27-2803.07) | 2,162 (23.5) | 95 (21.4) | 0.97 (0.71-1.33) | 28 (17.4) | 0.89 (0.51-1.56) | 37,042 (25.1) | 523 (24.6) | 1.03 (0.90-1.17) | 78 (24.5) | 1.36 (0.95-1.94) |
| Q4 (≥ 2803.08) | 2,072 (22.5) | 88 (19.8) | Reference | 27 (16.8) | Reference | 37,201 (25.2) | 486 (22.8) | Reference | 54 (16.9) | Reference |
| P-interaction | | | 0.28 | | 0.08 | | | 0.28 | | 0.08 |
| **Iron (mg/day)** | | | | | | | | | | |
| Q1 (< 6.93) | 2,471 (26.9) | 141 (31.8) | 1.05 (0.80-1.38) | 70 (43.5) | **1.62 (1.06-2.48)** | 36,521 (24.8) | 612 (28.8) | 1.02 (0.91-1.16) | 112 (35.1) | **1.46 (1.07-2.00)** |
| Q2 (6.94-9.16) | 2,305 (25.0) | 111 (25.0) | Reference | 39 (24.2) | Reference | 36,855 (25.0) | 548 (25.7) | Reference | 70 (21.9) | Reference |
| Q3 (9.17-12.12) | 2,228 (24.2) | 95 (21.4) | 1.03 (0.75-1.44) | 25 (15.5) | 0.73 (0.41-1.30) | 36,974 (25.1) | 523 (24.6) | 1.01 (0.87-1.16) | 83 (26.0) | 1.25 (0.87-1.79) |
| Q4 (≥ 12.13) | 2,196 (23.9) | 97 (21.8) | 1.20 (0.83-1.73) | 27 (16.8) | 0.86 (0.46-1.62) | 37,108 (25.2) | 446 (20.9) | 0.89 (0.76-1.05) | 54 (16.9) | 0.86 (0.56-1.33) |
| P-interaction | | | 0.43 | | **< 0.01** | | | 0.43 | | **< 0.01** |
| **Zinc (mg/day)** | | | | | | | | | | |
| Q1 (< 5.86) | 2,481 (27.0) | 139 (31.3) | 0.94 (0.64-1.38) | 67 (41.6) | ***1.94 (0.99-3.80)*** | 36,539 (24.8) | 590 (27.7) | 1.00 (0.85-1.20) | 111 (34.8) | 1.31 (0.84-2.05) |
| Q2 (5.87-7.37) | 2,331 (25.3) | 112 (25.2) | 0.98 (0.69-1.41) | 41 (25.5) | 1.44 (0.78-2.65) | 36,804 (25.0) | 569 (26.7) | 1.04 (0.89-1.21) | 71 (22.3) | 0.91 (0.60-1.37) |
| Q3 (7.38-9.35) | 2,275 (24.7) | 92 (20.7) | Reference | 28 (17.4) | Reference | 36,952 (25.1) | 512 (24,0) | Reference | 69 (21.6) | Reference |
| Q4 (≥ 9.36) | 2,113 (23.0) | 101 (22.7) | **1.48 (1.08-2.03)** | 25 (15.5) | 1.04 (0.59-1.83) | 37,163 (25.2) | 458 (21.5) | 0.96 (0.84-1.09) | 68 (21.3) | 1.05 (0.74-1.49) |
| P-interaction | | | 0.46 | | **< 0.01** | | | 0.46 | | **< 0.01** |

[1] Chronic kidney disease (CKD) was defined as the eGFR (estimated GFR) of less than 60 mL/min/1.73 m2 on the basis of the National Kidney Foundation's Kidney Disease Outcome Quality Initiative working group definition (K/DOQI clinical practice guidelines for chronic kidney disease: evaluation, classification, and stratification. Am J Kidney Dis 2002;39 (suppl 1):S1–266.).

[2] Early stage CKD was defined as 45 ≤ eGFR < 60 ml/min/1.73 $m^2$ and advanced stage CKD was defined as eGFR (estimated GFR) of less than 45 mL/min/1.73 $m^2$.

[3] Adjusted for age, sex, energy intake per day, body mass index, regular exercise, smoking status, history of hypertension & diabetes, albumin, protein intake per day, use of dietary supplements, uric acid, and cholesterol.

[4] Hypertension was defined as a person with anti-hypertensive medication or systolic blood pressure ≥ 140, diastolic blood pressure ≥ 90 mmHg, or the presence of history of hypertension.

**Table S3.** Association between dietary mineral intake and CKD (using the CKD-EPI equation[1]) in the Health Examinees (HEXA) study of the Korea Genome and Epidemiologic Study (KoGES), 2005-2012

| Mineral intake | Non-CKD[1] (N=156,965) | Early stage CKD[2] (N=2,245) | | Advanced stage CKD[2] (N=501) | |
|---|---|---|---|---|---|
| | N (%) | N (%) | OR (95% CI)[3] | N (%) | OR (95% CI)[3] |
| **Calcium (mg/day)** | | | | | |
| Q1 (< 273.37) | 39,130 (25.0) | 629 (28.0) | 0.89 (0.76-1.04) | 168 (33.5) | 1.23 (0.88-1.71) |
| Q2 (273.38-401.36) | 39,247 (25.0) | 544 (24.2) | 0.88 (0.77-1.03) | 137 (27.3) | 1.18 (0.87-1.60) |
| Q3 (401.37-567.66) | 39,280 (25.0) | 543 (24.2) | 0.94 (0.82-1.07) | 105 (21.0) | 1.00 (0.75-1.34) |
| Q4 (≥ 567.67) | 39,308 (25.0) | 529 (23.6) | Reference | 91 (18.2) | Reference |
| **Phosphorus (mg/day)** | | | | | |
| Q1 (< 663.68) | 39,093 (24.9) | 643 (28.6) | 0.98 (0.87-1.11) | 191 (38.1) | **1.53 (1.19-1.95)** |
| Q2 (663.69-844.27) | 39,236 (25.0) | 576 (25.7) | Reference | 116 (23.1) | Reference |
| Q3 (844.28-1067.44) | 39,267 (25.0) | 552 (24.6) | 1.11 (0.94-1.31) | 109 (21.8) | 0.98 (0.68-1.40) |
| Q4 (≥ 1067.45) | 39,369 (25.1) | 474 (21.1) | 1.08 (0.89-1.32) | 85 (17.0) | 0.81 (0.53-1.24) |
| **Sodium (mg/day)** | | | | | |
| Q1 (< 1541.09) | 39,174 (25.0) | 600 (26.7) | 1.00 (0.88-1.13) | 153 (30.5) | 0.98 (0.77-1.25) |
| Q2 (1541.10-2350.69) | 39,238 (25.0) | 552 (24.6) | Reference | 138 (27.5) | Reference |
| Q3 (2350.70-3260.41) | 39,272 (25.0) | 539 (24.0) | 0.98 (0.87-1.11) | 117 (23.4) | 0.89 (0.70-1.15) |
| Q4 (≥ 3260.42) | 39,281 (25.0) | 554 (24.7) | 0.99 (0.87-1.12) | 93 (18.6) | 0.76 (0.57-1.00) |
| **Potassium (mg/day)** | | | | | |
| Q1 (< 1567.53) | 39,091 (24.9) | 644 (28.7) | 0.98 (0.83-1.15) | 192 (38.3) | **1.74 (1.22-2.47)** |
| Q2 (1567.54-2114.26) | 39,239 (25.0) | 572 (25.5) | 1.01 (0.87-1.17) | 117 (23.3) | 1.20 (0.86-1.67) |
| Q3 (2114.27-2803.07) | 39,277 (25.0) | 542 (24.1) | 1.04 (0.91-1.19) | 109 (21.8) | 1.20 (0.89-1.62) |
| Q4 (≥ 2803.08) | 39,358 (25.1) | 487 (21.7) | Reference | 83 (16.6) | Reference |
| **Iron (mg/day)** | | | | | |
| Q1 (< 6.93) | 39,073 (24.9) | 662 (29.5) | 1.03 (0.91-1.16) | 192 (38.3) | **1.56 (1.22-2.00)** |
| Q2 (6.94-9.16) | 39,242 (25.0) | 572 (25.5) | Reference | 114 (22.7) | Reference |
| Q3 (9.17-12.12) | 39,272 (25.0) | 542 (24.1) | 1.04 (0.91-1.19) | 114 (22.7) | 1.07 (0.80-1.45) |
| Q4 (≥ 12.13) | 39,378 (25.1) | 469 (20.9) | 0.96 (0.82-1.13) | 81 (16.2) | 0.80 (0.57-1.15) |
| **Zinc (mg/day)** | | | | | |
| Q1 (< 5.86) | 39,104 (24.9) | 635 (28.3) | 0.96 (0.81-1.14) | 188 (37.5) | **1.64 (1.13-2.37)** |
| Q2 (5.87-7.37) | 39,216 (25.0) | 595 (26.5) | 1.00 (0.86-1.16) | 117 (23.3) | 1.11 (0.79-1.55) |
| Q3 (7.38-9.35) | 39,297 (25.0) | 531 (23.6) | Reference | 100 (20.0) | Reference |
| Q4 (≥ 9.36) | 39,348 (25.1) | 484 (21.6) | 1.02 (0.89-1.16) | 96 (19.2) | 1.02 (0.76-1.37) |

[1] Chronic kidney disease (CKD) was defined as the eGFR (estimated GFR) of less than 60 mL/min/1.73 m$^2$ using the CKD epidemiology collaboration (CKD-EPI) creatinine equation [1].

[2] Early stage CKD was defined as 45 ≤ eGFR < 60 ml/min/1.73 m$^2$ and advanced stage CKD was defined as eGFR (estimated GFR) of less than 45 mL/min/1.73 m$^2$.

[3] Adjusted for age, sex, energy intake per day, body mass index, regular exercise, smoking status, history of hypertension & diabetes, albumin, protein intake per day, use of dietary supplements, uric acid, and cholesterol.

**Table S4.** Association between dietary mineral intake and CKD (using the Asian modified CKD-EPI equation[1]) in the Health Examinees (HEXA) study of the Korea Genome and Epidemiologic Study (KoGES), 2005-2012

| **Mineral intake** | **Non-CKD[1] (N=157,939)** | **Early stage CKD[2] (N=1,344)** | | **Advanced stage CKD[2] (N=428)** | |
|---|---|---|---|---|---|
| | N (%) | N (%) | OR (95% CI)[3] | N (%) | OR (95% CI)[3] |
| **Calcium (mg/day)** | | | | | |
| Q1 (< 273.37) | 39,396 (25.0) | 389 (28.9) | 0.91 (0.74-1.10) | 142 (33.2) | 1.28 (0.89-1.84) |
| Q2 (273.38-401.36) | 39,486 (25.0) | 320 (23.8) | 0.86 (0.71-1.03) | 122 (28.5) | 1.30 (0.93-1.81) |
| Q3 (401.37-567.66) | 39,519 (25.0) | 319 (23.7) | 0.91 (0.77-1.08) | 90 (21.0) | 1.06 (0.77-1.46) |
| Q4 (≥ 567.67) | 39,538 (25.0) | 316 (23.5) | Reference | 74 (17.3) | Reference |
| **Phosphorus (mg/day)** | | | | | |
| Q1 (< 663.68) | 39,366 (24.9) | 395 (29.4) | 1.01 (0.86-1.17) | 166 (38.8) | **1.56 (1.19-2.03)** |
| Q2 (663.69-844.27) | 39,478 (25.0) | 351 (26.1) | Reference | 99 (23.1) | Reference |
| Q3 (844.28-1067.44) | 39,518 (25.0) | 318 (23.7) | 0.97 (0.78-1.19) | 92 (21.5) | 0.99 (0.67-1.47) |
| Q4 (≥ 1067.45) | 39,577 (25.1) | 280 (20.8) | 0.95 (0.74-1.22) | 71 (16.6) | 0.81 (0.51-1.28) |
| **Sodium (mg/day)** | | | | | |
| Q1 (< 1541.09) | 39,450 (25.0) | 350 (26.0) | 0.93 (0.79-1.09) | 127 (29.7) | 0.94 (0.72-1.21) |
| Q2 (1541.10-2350.69) | 39,468 (25.0) | 342 (25.4) | Reference | 118 (27.6) | Reference |
| Q3 (2350.70-3260.41) | 39,521 (25.0) | 301 (22.4) | 0.89 (0.76-1.04) | 106 (24.8) | 0.96 (0.73-1.25) |
| Q4 (≥ 3260.42) | 39,500 (25.0) | 351 (26.1) | 1.04 (0.88-1.22) | 77 (18.0) | 0.75 (0.55-1.02) |
| **Potassium (mg/day)** | | | | | |
| Q1 (< 1567.53) | 39,370 (24.9) | 391 (29.1) | 1.03 (0.83-1.27) | 166 (38.8) | **1.71 (1.17-2.51)** |
| Q2 (1567.54-2114.26) | 39,477 (25.0) | 355 (26.4) | 1.08 (0.89-1.31) | 96 (22.4) | 1.14 (0.79-1.63) |
| Q3 (2114.27-2803.07) | 39,518 (25.0) | 314 (23.4) | 1.03 (0.87-1.22) | 96 (22.4) | 1.25 (0.91-1.72) |
| Q4 (≥ 2803.08) | 39,574 (25.1) | 284 (21.1) | Reference | 70 (16.4) | Reference |
| **Iron (mg/day)** | | | | | |
| Q1 (< 6.93) | 39,359 (24.9) | 404 (30.1) | 1.05 (0.90-1.22) | 164 (38.3) | **1.53 (1.17-2.00)** |
| Q2 (6.94-9.16) | 39,484 (25.0) | 347 (25.8) | Reference | 97 (22.7) | Reference |
| Q3 (9.17-12.12) | 39,518 (25.0) | 313 (23.3) | 0.97 (0.81-1.16) | 97 (22.7) | 1.12 (0.81-1.54) |
| Q4 (≥ 12.13) | 39,578 (25.1) | 280 (20.8) | 0.93 (0.76-1.14) | 70 (16.4) | 0.87 (0.59-1.27) |
| **Zinc (mg/day)** | | | | | |
| Q1 (< 5.86) | 39,373 (24.9) | 388 (28.9) | 0.93 (0.75-1.15) | 166 (38.8) | **1.86 (1.24-2.79)** |
| Q2 (5.87-7.37) | 39,487 (25.0) | 341 (25.4) | 0.91 (0.75-1.10) | 100 (23.4) | 1.20 (0.83-1.73) |
| Q3 (7.38-9.35) | 39,518 (25.0) | 329 (24.5) | Reference | 81 (18.9) | Reference |
| Q4 (≥ 9.36) | 39,561 (25.1) | 286 (21.3) | 0.98 (0.83-1.16) | 81 (18.9) | 1.05 (0.76-1.45) |

[1] Chronic kidney disease (CKD) was defined as the eGFR (estimated GFR) of less than 60 mL/min/1.73 m² using the Asian modified CKD epidemiology collaboration (CKD-EPI) creatinine equation

[2] Early stage CKD was defined as 45 ≤ eGFR < 60 ml/min/1.73 m² and advanced stage CKD was defined as eGFR (estimated GFR) of less than 45 mL/min/1.73 m².

[3] Adjusted for age, sex, energy intake per day, body mass index, regular exercise, smoking status, history of hypertension & diabetes, albumin, protein intake per day, use of dietary supplements, uric acid, and cholesterol.

**Table S5.** Association between dietary mineral intake and protein intake status in the Health Examinees (HEXA) study of the Korea Genome and Epidemiologic Study (KoGES), 2005-2012

| Mineral intake | Low protein intake[1] (N=79,856) | High protein intake[2] (N=79,855) | |
|---|---|---|---|
| | N (%) | N (%) | P-value[3] |
| **Calcium (mg/day)** | | | |
| Q1 (< 273.37) | 36,801 (46.1) | 3,126 (3.9) | < 0.01 |
| Q2 (273.38-401.36) | 25,809 (32.3) | 14,119 (17.7) | < 0.01 |
| Q3 (401.37-567.66) | 14,141 (17.7) | 25,787 (32.3) | < 0.01 |
| Q4 (≥ 567.67) | 3,105 (3.9) | 36,823 (46.1) | Reference |
| **Phosphorus (mg/day)** | | | |
| Q1 (< 663.68) | 39,900 (50.0) | 27 (0.1) | < 0.01 |
| Q2 (663.69-844.27) | 33,471 (41.9) | 6,457 (8.1) | Reference |
| Q3 (844.28-1067.44) | 6,438 (8.1) | 33,490 (41.9) | < 0.01 |
| Q4 (≥ 1067.45) | 47 (0.1) | 39,881 (49.9) | < 0.01 |
| **Sodium (mg/day)** | | | |
| Q1 (< 1541.09) | 34,257 (42.9) | 5,670 (7.1) | < 0.01 |
| Q2 (1541.10-2350.69) | 23,111 (28.9) | 16,817 (21.1) | Reference |
| Q3 (2350.70-3260.41) | 15,947 (20.0) | 23,981 (30.0) | < 0.01 |
| Q4 (≥ 3260.42) | 6,541 (8.2) | 33,387 (41.8) | < 0.01 |
| **Potassium (mg/day)** | | | |
| Q1 (< 1567.53) | 38,024 (47.6) | 1,903 (2.4) | < 0.01 |
| Q2 (1567.54-2114.26) | 27,337 (34.2) | 12,591 (15.8) | < 0.01 |
| Q3 (2114.27-2803.07) | 12,139 (15.2) | 27,789 (34.8) | < 0.01 |
| Q4 (≥ 2803.08) | 2,356 (2.9) | 375,72 (47.1) | Reference |
| **Iron (mg/day)** | | | |
| Q1 (< 6.93) | 39,303 (49.2) | 624 (0.8) | < 0.01 |
| Q2 (6.94-9.16) | 29,298 (36.7) | 10,630 (13.3) | Reference |
| Q3 (9.17-12.12) | 9,809 (12.3) | 30,119 (37.7) | < 0.01 |
| Q4 (≥ 12.13) | 1,446 (1.8) | 38,482 (48.2) | < 0.01 |
| **Zinc (mg/day)** | | | |
| Q1 (< 5.86) | 39,577 (49.6) | 350 (0.4) | < 0.01 |
| Q2 (5.87-7.37) | 31,326 (39.2) | 8,602 (10.8) | < 0.01 |
| Q3 (7.38-9.35) | 8,156 (10.2) | 31,772 (39.8) | Reference |
| Q4 (≥ 9.36) | 797 (1.0) | 39,131 (49.0) | < 0.01 |

[1] Chronic kidney disease (CKD) was defined as the eGFR (estimated GFR) of less than 60 mL/min/1.73 $m^2$ on the basis of the National Kidney Foundation's Kidney Disease Outcome Quality Initiative working group definition (K/DOQI clinical practice guidelines for chronic kidney disease: evaluation, classification, and stratification. Am J Kidney Dis 2002;39(suppl 1):S1–266.).

[2] Low protein levels: 55.27g/day, High protein levels: ≥ 55.27g/day – median

[3] Adjusted for age, sex, energy intake per day, body mass index, regular exercise, smoking status, history of hypertension & diabetes, albumin, use of dietary supplements, uric acid, and cholesterol.

**Table S6.** General characteristics of study population stratified by each dietary mineral intake[1] status in the Health Examinees Study (HEXA) of a major urban cohort in the Korea Genome and Epidemiologic Study (KoGES), 2005-2012

| | **Dietary calcium intake (mg/day)** | | | | | **Dietary phosphorus intake (mg/day)** | | | | |
|---|---|---|---|---|---|---|---|---|---|---|
| **Mineral intake** | **1Q (N=39,927)** | **2Q (N=39,928)** | **3Q (N=39,928)** | **4Q (N=39,928)** | ***p*-value** | **1Q (N=39,927)** | **2Q (N=39,928)** | **3Q (N=39,928)** | **4Q (N=39,928)** | ***p*-value** |
| | **N (%)** | **N (%)** | **N (%)** | **N (%)** | | **N (%)** | **N (%)** | **N (%)** | **N (%)** | |
| Sex | | | | | | | | | | |
| Male | 14,689 (27.3) | 14,364 (26.7) | 13,215 (24.5) | 11,612 (21.5) | **<0.01** | 11,404 (21.2) | 13,688 (25.4) | 14,299 (26.5) | 14,489 (26.9) | **<0.01** |
| Female | 25,238 (23.8) | 25,564 (24.2) | 26,713 (25.2) | 28,316 (26.8) | | 28,523 (26.9) | 26,240 (24.8) | 25,629 (24.2) | 25,439 (24.0) | |
| Marital status | | | | | | | | | | |
| Single | 5,008 (29.3) | 4,169 (24.4) | 4,014 (10.0) | 3,899 (9.8) | **<0.01** | 5,660 (33.1) | 4,357 (25.5) | 3,602 (21.1) | 3,471 (20.3) | **<0.01** |
| Married | 32,916 (24.5) | 33,848 (25.2) | 33,978 (25.3) | 33,573 (25.0) | | 32,217 (23.9) | 33,682 (25.1) | 34,406 (25.6) | 34,010 (25.3) | |
| Others | 2,003 (24.1) | 1,911 (23.0) | 1,936 (23.3) | 2,456 (29.6) | | 2,050 (24.7) | 1,889 (22.7) | 1,920 (23.1) | 2,447 (29.5) | |
| Education | | | | | | | | | | |
| Below Middle school | 8,846 (32.6) | 6,824 (25.1) | 6,224 (22.9) | 5,276 (19.4) | **<0.01** | 9,326 (34.3) | 7,266 (26.7) | 5,852 (21.5) | 4,726 (17.4) | **<0.01** |
| High school | 20,979 (24.0) | 21,735 (24.8) | 22,192 (25.3) | 22,650 (25.9) | | 21,115 (24.1) | 21,618 (24.7) | 22,244 (25.4) | 22,579 (25.8) | |
| Higher than College | 9,477 (22.2) | 10,853 (25.5) | 10,894 (25.6) | 11,401 (26.7) | | 8,878 (20.8) | 10,413 (24.4) | 11,237 (26.4) | 12,077 (28.3) | |
| Monthly household income (KRW) | | | | | | | | | | |
| < 1,500,000 | 5,114 (33.5) | 3,741 (24.5) | 3,446 (22.6) | 2,958 (19.4) | **<0.01** | 5,574 (36.5) | 3,995 (26.2) | 3,133 (20.5) | 2,557 (16.8) | **<0.01** |
| 1,500,000 - 2,999,999 | 7,149 (26.6) | 6,872 (25.6) | 6,566 (24.4) | 6,267 (23.3) | | 7,225 (26.9) | 7,035 (26.2) | 6,520 (24.3) | 6,074 (22.6) | |
| 3,000,000 – 3,999,999 | 13,090 (22.8) | 14,678 (25.6) | 14,976 (26.1) | 14,684 (25.6) | | 12,642 (22.0) | 14,205 (24.7) | 15,240 (26.5) | 15,341 (26.7) | |
| ≥ 4,000,000 | 7,732 (22.8) | 8,492 (25.1) | 8,647 (25.5) | 8,979 (26.5) | | 7,391 (21.8) | 8,353 (24.7) | 8,882 (26.2) | 9,224 (27.2) | |
| Regular exercise[3] | | | | | | | | | | |
| No | 21,758 (28.7) | 19,645 (25.9) | 18,225 (24.1) | 16,096 (21.3) | **<0.01** | 21,206 (28.0) | 19,410 (25.6) | 18,301 (24.2) | 16,807 (22.2) | **<0.01** |
| Yes | 18,006 (21.6) | 20,188 (24.2) | 21,517 (25.8) | 23,700 (28.4) | | 18,555 (22.2) | 20,357 (24.4) | 21,506 (25.8) | 22,993 (27.6) | |
| Drinking | | | | | | | | | | |
| No | 19,871 (24.8) | 19,443 (24.2) | 20,063 (25.0) | 20,789 (25.9) | **<0.01** | 21,844 (27.2) | 20,138 (25.1) | 19,393 (24.2) | 18,791 (23.4) | **<0.01** |
| Yes | 19,920 (25.2) | 20,360 (25.8) | 19,672 (24.9) | 18,930 (24.0) | | 17,943 (22.7) | 19,615 (24.9) | 20,389 (25.8) | 20,935 (26.5) | |
| Smoking | | | | | | | | | | |
| No | 15,390 (24.6) | 14,758 (23.6) | 15,448 (24.7) | 17,019 (27.2) | **<0.01** | 16,380 (26.2) | 15,283 (24.4) | 15,204 (24.3) | 15,748 (25.1) | **<0.01** |

| | | | | | | | | | | |
|---|---|---|---|---|---|---|---|---|---|---|
| Yes | 5,756 (27.3) | 5,623 (26.6) | 5,133 (24.3) | 4,597 (21.8) | | 4,553 (21.6) | 5,239 (24.8) | 5,629 (26.7) | 5,688 (26.9) | |
| Passive smoking[4] | | | | | | | | | | |
| No | 27,500 (25.0) | 27,759 (25.2) | 27,876 (25.3) | 26,960 (24.5) | **<0.01** | 27,938 (25.4) | 28,034 (25.5) | 27,823 (25.3) | 26,300 (23.9) | **<0.01** |
| Yes | 10,454 (25.4) | 10,247 (24.9) | 10,088 (24.5) | 10,411 (25.3) | | 9,954 (24.2) | 9,998 (24.3) | 10,152 (24.6) | 11,096 (26.9) | |
| Hypertension[5] | | | | | | | | | | |
| No | 27,891 (24.4) | 28,503 (24.9) | 28,828 (25.2) | 29,201 (25.5) | **<0.01** | 28,044 (24.5) | 28,390 (24.8) | 28,755 (25.1) | 29,234 (25.5) | **<0.01** |
| Yes | 12,036 (26.6) | 11,425 (25.2) | 11,100 (24.5) | 10,727 (23.7) | | 11,883 (26.2) | 11,538 (25.5) | 11,173 (24.7) | 10,694 (23.6) | |
| Diabetes[6] | | | | | | | | | | |
| No | 37,155 (24.8) | 37,485 (25.0) | 37,549 (25.0) | 37,717 (25.2) | **<0.01** | 37,172 (24.8) | 37,439 (25.0) | 37,610 (25.1) | 37,685 (25.1) | **<0.01** |
| Yes | 2,772 (28.3) | 2,443 (24.9) | 2,379 (24.3) | 2,211 (22.5) | | 2,755 (28.1) | 2,489 (25.4) | 2,318 (23.6) | 2,243 (22.9) | |
| Use of dietary supplements | | | | | | | | | | |
| No | 36,361 (25.5) | 36,072 (25.3) | 35,559 (24.9) | 34,517 (24.2) | **<0.01** | 36,161 (25.4) | 35,996 (25.3) | 25,452 (24.9) | 34,900 (24.5) | **<0.01** |
| Yes | 3,285 (20.5) | 3,612 (22.5) | 4,051 (25.2) | 5,101 (31.8) | | 3,470 (21.6) | 3,621 (22.6) | 4,230 (26.4) | 4,728 (29.5) | |
| | **Mean (SD)** | **Mean (SD)** | **Mean (SD)** | **Mean (SD)** | | **Mean (SD)** | **Mean (SD)** | **Mean (SD)** | **Mean (SD)** | |
| Age (year) | 53.4 (8.55) | 52.3 (8.37) | 52.5 (8.31) | 52.2 (8.10) | **<0.01** | 53.6 (8.48) | 52.7 (8.33) | 52.3 (8.30) | 51.6 (8.13) | **<0.01** |
| Height (cm) | 160.4 (8.24) | 160.8 (8.10) | 160.5 (7.99) | 160.3 (7.77) | **<0.01** | 159.2 (7.86) | 160.5 (8.01) | 160.9 (8.05) | 161.4 (8.04) | **<0.01** |
| Weight (kg) | 61.5 (10.00) | 62.1 (9.94) | 61.8 (9.95) | 61.7 (9.80) | **<0.01** | 60.3 (9.53) | 61.8 (9.80) | 62.2 (9.91) | 63.0 (10.25) | **<0.01** |
| BMI (kg/m$^2$) | 23.8 (2.95) | 23.9 (2.89) | 23.9 (2.92) | 23.9 (2.91) | **<0.01** | 23.7 (2.93) | 23.9 (2.89) | 23.9 (2.87) | 24.1 (2.94) | **<0.01** |
| Albumin (g/dL) | 4.6 (0.28) | 4.6 (0.27) | 4.6 (0.27) | 4.6 (0.27) | **<0.01** | 4.6 (0.28) | 4.6 (0.27) | 4.6 (0.27) | 4.6 (0.27) | **<0.01** |
| Protein intake (g) | 39.4 (11.37) | 52.4 (13.07) | 62.4 (15.82) | 86.9 (35.42) | **<0.01** | 35.5 (7.20) | 49.8 (5.61) | 62.5 (7.27) | 93.3 (32.55) | **<0.01** |
| Creatinine (mg/dL) | 0.8 (0.23) | 0.8 (0.22) | 0.8 (0.19) | 0.8 (0.22) | **<0.01** | 0.8 (0.23) | 0.8 (0.21) | 0.8 (0.22) | 0.8 (0.21) | **<0.01** |
| Uric acid (mg/dL) | 4.7 (1.31) | 4.7 (1.30) | 4.7 (1.28) | 4.6 (1.24) | **<0.01** | 4.6 (1.26) | 4.7 (1.28) | 4.7 (1.29) | 4.8 (1.29) | **<0.01** |
| Total cholesterol (mg/dL) | 196.4 (35.45) | 196.9 (35.00) | 198.6 (35.33) | 199.5 (35.36) | **<0.01** | 197.1 (35.57) | 197.4 (35.13) | 198.2 (35.13) | 198.9 (35.37) | **<0.01** |

[1] Chronic kidney disease (CKD) was defined as the eGFR (estimated GFR) of less than 60 mL/min/1.73 m$^2$ on the basis of the National Kidney Foundation's Kidney Disease Outcome Quality Initiative working group definition (K/DOQI clinical practice guidelines for chronic kidney disease: evaluation, classification, and stratification. Am J Kidney Dis 2002;39(suppl 1):S1–266.)
[2] Patients with chronic kidney disease, cardiovascular disease and severe cancers (stomach cancer, liver cancer, colon cancer, lung cancer) are excluded from the study population. eGFR was calculated by MDRD formula [eGFR: 186×serum creatinine (mg/dL)$^{-1.154}$×age$^{-0.203}$×(0.742 if female)].
[3] Regular exercise was defined as performing regular exercise enough to sweat once a week or more.
[4] Passive smoking (among subjects who had never smoked) was determined by asking. "How many times do you indirectly inhale smoke from other people at home or your workplace?"
[5] Hypertension was defined as a person with anti-hypertensive medication or systolic blood pressure ≥ 140, diastolic blood pressure ≥ 90 mmHg, or the presence of history of hypertension.
[6] Diabetes was defined as fasting blood glucose ≥ 126 mg/ml or the presence of history of diabetes.

**Table S7.** General characteristics of study population stratified by each dietary mineral intake[1] status in the Health Examinees Study (HEXA) of a major urban cohort in the Korea Genome and Epidemiologic Study (KoGES), 2005-2012

| | Dietary Sodium intake (mg/day) | | | | | Dietary Potassium intake (mg/day) | | | | |
|---|---|---|---|---|---|---|---|---|---|---|
| **Mineral intake** | **1Q (N=39,927)** | **2Q (N=39,928)** | **3Q (N=39,928)** | **4Q (N=39,928)** | ***p*-value** | **1Q (N=39,927)** | **2Q (N=39,928)** | **3Q (N=39,928)** | **4Q (N=39,928)** | ***p*-value** |
| | **N (%)** | **N (%)** | **N (%)** | **N (%)** | | **N (%)** | **N (%)** | **N (%)** | **N (%)** | |
| Sex | | | | | | | | | | |
| Male | 11,434 (21.2) | 12,857 (23.9) | 14,008 (26.0) | 15,581 (28.9) | **<0.01** | 13,020 (24.2) | 13,844 (25.7) | 13,839 (25.7) | 13,177 (24.5) | **<0.01** |
| Female | 28,493 (26.9) | 27,071 (25.6) | 25,920 (24.5) | 24,347 (23.0) | | 26,907 (25.4) | 26,084 (24.6) | 26,089 (24.6) | 26,751 (25.3) | |
| Marital status | | | | | | | | | | |
| Single | 5,277 (30.9) | 4,404 (25.8) | 3,781 (22.1) | 3,628 (21.2) | **<0.01** | 5,557 (32.5) | 4,304 (25.2) | 3,763 (22.0) | 3,466 (20.3) | **<0.01** |
| Married | 32,636 (24.3) | 33,637 (25.0) | 34,365 (25.6) | 33,677 (25.1) | | 32,375 (24.1) | 33,793 (25.2) | 34,295 (25.5) | 33,852 (25.2) | |
| Others | 2,014 (24.2) | 1,887 (22.7) | 1,782 (21.4) | 2,623 (31.6) | | 1,995 (24.0) | 1,831 (22.0) | 1,870 (22.5) | 2,610 (31.4) | |
| Education | | | | | | | | | | |
| Below Middle school | 7,636 (28.1) | 6,634 (24.4) | 6,551 (24.1) | 6,349 (23.4) | **<0.01** | 9,269 (34.1) | 7,146 (26.3) | 5,953 (21.9) | 4,802 (17.7) | **<0.01** |
| High school | 21,805 (24.9) | 21,610 (24.7) | 21,956 (25.1) | 22,185 (25.3) | | 21,078 (24.1) | 21,643 (24.7) | 22,216 (25.4) | 22,619 (25.8) | |
| Higher than College | 9,930 (23.3) | 11,134 (26.1) | 10,823 (25.4) | 10,738 (25.2) | | 8,961 (21.0) | 10,517 (24.7) | 11,224 (26.3) | 11,923 (28.0) | |
| Monthly household income (KRW) | | | | | | | | | | |
| < 1,500,000 | 4,664 (30.6) | 3,746 (24.5) | 3,511 (23.0) | 3,338 (21.9) | **<0.01** | 5,479 (35.9) | 3,958 (25.9) | 3,161 (20.7) | 2,661 (17.4) | **<0.01** |
| 1,500,000 - 2,999,999 | 7,022 (26.1) | 6,605 (24.6) | 6,795 (25.3) | 6,432 (23.9) | | 7,409 (27.6) | 6,968 (25.9) | 6.556 (24.4) | 5,921 (22.0) | |
| 3,000,000 – 3,999,999 | 13,654 (23.8) | 14,585 (25.4) | 14,960 (26.1) | 14,229 (24.8) | | 12,949 (22.5) | 14,324 (24.9) | 15,286 (26.6) | 14,869 (25.9) | |
| ≥ 4,000,000 | 8,172 (24.1) | 8,944 (26.4) | 8,552 (25.3) | 8,182 (24.2) | | 7,205 (21.3) | 8,491 (25.1) | 8,834 (26.1) | 9,320 (27.5) | |
| Regular exercise[3] | | | | | | | | | | |
| No | 19,511 (25.8) | 19,130 (25.3) | 18,795 (24.8) | 19,288 (24.1) | **<0.01** | 21,340 (28.2) | 19,674 (26.0) | 28,433 (24.3) | 16,277 (21.5) | **<0.01** |
| Yes | 20,248 (24.3) | 20,687 (24.8) | 20,956 (25.1) | 21,520 (25.8) | | 18,410 (22.1) | 20,095 (24.1) | 21,386 (25.6) | 23,520 (28.2) | |
| Drinking | | | | | | | | | | |
| No | 21,710 (27.1) | 20,178 (25.2) | 19,815 (24.7) | 18,463 (23.0) | **<0.01** | 21,108 (26.3) | 19,956 (24.9) | 19,550 (24.4) | 19,552 (24.4) | **<0.01** |
| Yes | 18,058 (22.9) | 19,617 (24.9) | 19,922 (25.3) | 21,285 (27.0) | | 18,680 (23.7) | 19,795 (25.1) | 20,231 (25.7) | 20,176 (25.6) | |
| Smoking | | | | | | | | | | |
| No | 16,328 (26.1) | 14,826 (23.7) | 14,835 (23.7) | 16,626 (26.5) | **<0.01** | 15,646 (25.0) | 14,791 (23.6) | 15,233 (24.3) | 16,945 (27.1) | **<0.01** |
| Yes | 4,536 (21.5) | 4,739 (22.3) | 5,234 (24.8) | 6,600 (31.3) | | 4,991 (23.6) | 5,369 (25.4) | 5,394 (25.5) | 5,355 (25.4) | |

| | | | | | | | | | | |
|---|---|---|---|---|---|---|---|---|---|---|
| Passive smoking[4] | | | | | | | | | | |
| No | 27,742 (25.2) | 28,096 (25.5) | 28,020 (25.4) | 26,237 (23.8) | **<0.01** | 27,885 (25.3) | 28,042 (25.5) | 27,812 (25.3) | 26,356 (23.9) | **<0.01** |
| Yes | 10,158 (24.7) | 9,955 (24.2) | 10,069 (24.4) | 11,018 (26.7) | | 10,056 (24.4) | 10,051 (24.4) | 10,206 (24.8) | 10,887 (26.4) | |
| Hypertension[5] | | | | | | | | | | |
| No | 28,612 (25.0) | 28,847 (25.2) | 28,555 (25.0) | 28,409 (24.8) | **<0.01** | 27,846 (24.3) | 28,526 (24.9) | 28,973 (25.3) | 29,078 (25.4) | **<0.01** |
| Yes | 11,315 (25.0) | 11,081 (24.5) | 11,373 (25.1) | 11,519 (25.4) | | 12,081 (26.7) | 11,402 (25.2) | 10,955 (24.2) | 10,850 (24.0) | |
| Diabetes[6] | | | | | | | | | | |
| No | 37,336 (24.9) | 37,478 (25.0) | 37,572 (25.1) | 37,520 (25.0) | **<0.01** | 37,045 (24.7) | 37,477 (25.0) | 37,643 (25.1) | 37,741 (25.2) | **<0.01** |
| Yes | 2,591 (26.4) | 2,450 (25.0) | 2,356 (24.0) | 2,408 (24.6) | | 2,882 (29.4) | 2,451 (25.0) | 2,285 (23.3) | 2,187 (22.3) | |
| Use of dietary supplements | | | | | | | | | | |
| No | 35,727 (25.1) | 35,803 (25.1) | 35,746 (25.1) | 25,233 (24.7) | **<0.01** | 36,285 (25.5) | 36,049 (25.3) | 35,552 (24.9) | 34,623 (24.3) | **<0.01** |
| Yes | 3,916 (24.4) | 3,881 (24.2) | 3,879 (24.2) | 4,373 (27.2) | | 3,354 (20.9) | 3,570 (22.2) | 4,143 (25.8) | 4,982 (31.0) | |
| | **Mean (SD)** | **Mean (SD)** | **Mean (SD)** | **Mean (SD)** | | **Mean (SD)** | **Mean (SD)** | **Mean (SD)** | **Mean (SD)** | |
| Age (year) | 53.1 (8.31) | 52.5 (8.33) | 52.5 (8.31) | 52.1 (8.38) | **<0.01** | 53.6 (8.54) | 52.6 (8.40) | 52.2 (8.25) | 51.8 (8.07) | **<0.01** |
| Height (cm) | 159.6 (7.78) | 160.4 (7.94) | 160.7 (8.08) | 161.4 (8.23) | **<0.01** | 159.7 (8.08) | 160.6 (8.07) | 160.8 (8.00) | 161.0 (7.92) | **<0.01** |
| Weight (kg) | 60.5 (9.53) | 61.5 (9.83) | 62.1 (9.95) | 63.1 (10.19) | **<0.01** | 60.8 (9.78) | 61.7 (9.85) | 62.1 (9.96) | 62.5 (10.03) | **<0.01** |
| BMI (kg/m$^2$) | 23.7 (2.89) | 23.8 (2.90) | 23.9 (2.92) | 24.2 (2.93) | **<0.01** | 23.7 (2.95) | 23.9 (2.89) | 24.0 (2.90) | 24.1 (2.91) | **<0.01** |
| Albumin (g/dL) | 4.6 (0.27) | 4.6 (0.27) | 4.6 (0.27) | 4.7 (0.27) | **<0.01** | 4.6 (0.28) | 4.6 (0.27) | 4.6 (0.27) | 4.6 (0.27) | **<0.01** |
| Protein intake (g) | 42.1 (13.18) | 54.1 (15.96) | 62.1 (18.67) | 82.8 (36.87) | **<0.01** | 38.2 (9.92) | 51.2 (10.71) | 62.8 (13.58) | 88.8 (35.08) | **<0.01** |
| Creatinine (mg/dL) | 0.8 (0.22) | 0.8 (0.21) | 0.8 (0.22) | 0.8 (0.21) | **<0.01** | 0.8 (0.24) | 0.8 (0.22) | 0.8 (0.20) | 0.8 (0.21) | **0.02** |
| Uric acid (mg/dL) | 4.6 (1.26) | 4.7 (1.28) | 4.7 (1.29) | 4.8 (1.31) | **<0.01** | 4.7 (1.28) | 4.7 (1.29) | 4.7 (1.29) | 4.7 (1.29) | **<0.01** |
| Total cholesterol (mg/dL) | 197.2 (35.27) | 198.0 (35.44) | 198.3 (25.30) | 198.0 (35.20) | **<0.01** | 196.9 (35.50) | 197.6 (25.19) | 198.3 (35.25) | 198.8 (35.25) | **<0.01** |

[1] Chronic kidney disease (CKD) was defined as the eGFR (estimated GFR) of less than 60 mL/min/1.73 m$^2$ on the basis of the National Kidney Foundation's Kidney Disease Outcome Quality Initiative working group definition (K/DOQI clinical practice guidelines for chronic kidney disease: evaluation, classification, and stratification. Am J Kidney Dis 2002;39(suppl 1):S1–266.)

[2] Patients with chronic kidney disease, cardiovascular disease and severe cancers (stomach cancer, liver cancer, colon cancer, lung cancer) are excluded from the study population. eGFR was calculated by MDRD formula [eGFR: 186×serum creatinine (mg/dL)$^{-1.154}$×age$^{-0.203}$×(0.742 if female)].

[3] Regular exercise was defined as performing regular exercise enough to sweat once a week or more.

[4] Passive smoking (among subjects who had never smoked) was determined by asking. "How many times do you indirectly inhale smoke from other people at home or your workplace?"

[5] Hypertension was defined as a person with anti-hypertensive medication or systolic blood pressure ≥ 140, diastolic blood pressure ≥ 90 mmHg, or the presence of history of hypertension.

[6] Diabetes was defined as fasting blood glucose ≥ 126 mg/ml or the presence of history of diabetes.

**Table S8.** General characteristics of study population stratified by each dietary mineral intake[1] status in the Health Examinees Study (HEXA) of a major urban cohort in the Korea Genome and Epidemiologic Study (KoGES), 2005-2012

| | **Dietary Iron intake (mg/day)** | | | | | **Dietary Zinc intake (mg/day)** | | | | |
|---|---|---|---|---|---|---|---|---|---|---|
| **Mineral intake** | **1Q (N=39,927)** | **2Q (N=39,928)** | **3Q (N=39,928)** | **4Q (N=39,928)** | ***p*-value** | **1Q (N=39,927)** | **2Q (N=39,928)** | **3Q (N=39,928)** | **4Q (N=39,928)** | ***p*-value** |
| | **N (%)** | **N (%)** | **N (%)** | **N (%)** | | **N (%)** | **N (%)** | **N (%)** | **N (%)** | |
| Sex | | | | | | | | | | |
| Male | 12,500 (23.2) | 13,775 (25.6) | 13,893 (25.8) | 13,712 (25.4) | **<0.01** | 10,430 (19.4) | 13,201 (24.5) | 14,365 (26.7) | 15,884 (29.5) | **<0.01** |
| Female | 27,427 (25.9) | 26,153 (24.7) | 26,035 (24.6) | 26,216 (24.8) | | 29,497 (27.9) | 26,727 (25.2) | 25,563 (24.1) | 24,044 (22.7) | |
| Marital status | | | | | | | | | | |
| Single | 5,780 (14.5) | 4,241 (24.8) | 3,647 (21.3) | 3,422 (20.0) | **<0.01** | 5,838 (34.2) | 4,305 (25.2) | 3,633 (21.3) | 3,314 (19.4) | **<0.01** |
| Married | 32,108 (23.9) | 33,846 (25.2) | 34,409 (25.6) | 33,952 (25.3) | | 32,072 (23.9) | 33,649 (25.0) | 34,414 (25.6) | 34,180 (25.4) | |
| Others | 2,039 (24.5) | 1,841 (22.2) | 1,872 (22.5) | 2,554 (30.7) | | 2,017 (24.3) | 1,974 (23.8) | 1,881 (22.6) | 2,434 (29.3) | |
| Education | | | | | | | | | | |
| Below Middle school | 9,465 (34.8) | 7,236 (26.6) | 5,714 (21.0) | 4,755 (17.5) | **<0.01** | 9,505 (35.0) | 7,256 (26.7) | 5,852 (21.5) | 4,557 (16.8) | **<0.01** |
| High school | 21,085 (24.1) | 21,714 (24.8) | 22,311 (25.5) | 22,446 (25.6) | | 21,360 (24.4) | 21,745 (24.8) | 22,166 (25.3) | 22,285 (25.4) | |
| Higher than College | 8,743 (20.5) | 10,354 (24.3) | 11,364 (26.7) | 12,164 (28.5) | | 8,437 (19.8) | 10,288 (24.1) | 11,348 (26.6) | 12,552 (29.4) | |
| Monthly household income (KRW) | | | | | | | | | | |
| < 1,500,000 | 5,606 (36.7) | 3,922 (25.7) | 3,061 (20.1) | 2,670 (17.5) | **<0.01** | 5,740 (37.6) | 4,024 (26.4) | 3,112 (20.4) | 2,383 (15.6) | **<0.01** |
| 1,500,000 - 2,999,999 | 7,453 (27.7) | 7,006 (26.1) | 6,403 (23.8) | 5,992 (22.3) | | 7,359 (27.4) | 7,022 (26.1) | 6,519 (24.3) | 5,954 (22.2) | |
| 3,000,000 – 3,999,999 | 12,675 (22.1) | 14,231 (24.8) | 15,651 (27.2) | 14,871 (25.9) | | 12,676 (22.1) | 14,121 (24.6) | 15,021 (26.2) | 15,610 (27.2) | |
| ≥ 4,000,000 | 7,169 (21.2) | 8,460 (25.0) | 8,719 (25.8) | 9,502 (28.1) | | 6,996 (20.7) | 8,228 (24.3) | 9,067 (26.8) | 9,559 (28.2) | |
| Regular exercise[3] | | | | | | | | | | |
| No | 21,600 (28.5) | 19,530 (25.8) | 18,120 (23.9) | 16,474 (21.8) | **<0.01** | 21,229 (28.0) | 19,326 (25.5) | 18,170 (24.0) | 16,999 (22.4) | **<0.01** |
| Yes | 18,146 (21.7) | 20,241 (24.3) | 21,713 (26.0) | 23,311 (27.9) | | 18,522 (22.2) | 20,439 (24.5) | 21,654 (26.0) | 22,796 (27.3) | |
| Drinking | | | | | | | | | | |
| No | 21,140 (26.4) | 19,974 (24.9) | 19,654 (24.5) | 19,398 (24.2) | **<0.01** | 22,296 (27.8) | 20,577 (25.7) | 19,395 (24.2) | 17,898 (22.3) | **<0.01** |
| Yes | 18,637 (23.6) | 19,788 (25.1) | 20,139 (25.5) | 20,318 (25.8) | | 17,481 (22.2) | 19,170 (24.3) | 20,394 (25.8) | 21,837 (27.7) | |
| Smoking | | | | | | | | | | |
| No | 16,150 (25.8) | 15,200 (24.3) | 15,084 (24.1) | 16,181 (25.8) | **<0.01** | 16,656 (26.6) | 15,672 (25.0) | 15,257 (24.4) | 15,030 (24.0) | **<0.01** |
| Yes | 5,032 (23.8) | 5,307 (25.1) | 5,505 (26.1) | 5,265 (24.9) | | 4,299 (20.4) | 5,067 (24.0) | 5,615 (26.6) | 6,128 (29.0) | |

| | | | | | | | | | | |
|---|---|---|---|---|---|---|---|---|---|---|
| Passive smoking[4] | | | | | | | | | | |
| No | 27,691 (25.1) | 28,049 (25.5) | 27,892 (25.3) | 26,463 (24.0) | **<0.01** | 27,869 (25.3) | 28,058 (25.5) | 27,756 (25.2) | 26,412 (24.0) | **<0.01** |
| Yes | 10,209 (24.8) | 10,022 (24.3) | 10,149 (24.6) | 10,820 (26.3) | | 10,053 (24.4) | 9,873 (24.0) | 10,257 (24.9) | 11,017 (26.7) | |
| Hypertension[5] | | | | | | | | | | |
| No | 27,987 (24.5) | 28,531 (24.9) | 28,722 (25.1) | 29,183 (25.5) | **<0.01** | 28,133 (24.6) | 28,470 (24.9) | 28,671 (25.1) | 29,149 (25.5) | **<0.01** |
| Yes | 11,940 (26.4) | 11,397 (25.2) | 11,206 (24.7) | 10,745 (23.7) | | 11,794 (26.0) | 11,458 (25.3) | 11,257 (24.9) | 10,779 (23.8) | |
| Diabetes[6] | | | | | | | | | | |
| No | 37,245 (24.8) | 37,473 (25.0) | 37,580 (25.1) | 37,608 (25.1) | **<0.01** | 37,240 (24.8) | 37,444 (24.9) | 37,533 (25.0) | 37,689 (25.1) | **<0.01** |
| Yes | 2,682 (27.3) | 2,455 (25.0) | 2,348 (23.9) | 2,320 (23.7) | | 2,687 (27.4) | 22,484 (25.3) | 2,395 (24.4) | 2,239 (22.8) | |
| Use of dietary supplements | | | | | | | | | | |
| No | 36,198 (25.4) | 35,958 (25.2) | 35,603 (25.0) | 34,750 (24.4) | **<0.01** | 36,181 (25.4) | 35,875 (25.2) | 35,482 (24.9) | 34,971 (24.5) | **<0.01** |
| Yes | 3,408 (21.2) | 3,688 (23.0) | 4,087 (25.5) | 4,866 (30.3) | | 3,441 (21.4) | 3,741 (23.3) | 4,196 (26.1) | 4,671 (29.1) | |
| | **Mean (SD)** | **Mean (SD)** | **Mean (SD)** | **Mean (SD)** | | **Mean (SD)** | **Mean (SD)** | **Mean (SD)** | **Mean (SD)** | |
| Age (year) | 53.5 (8.53) | 52.7 (8.36) | 52.2 (8.26) | 51.8 (8.10) | **<0.01** | 53.4 (8.50) | 52.8 (8.35) | 52.3 (8.27) | 51.7 (8.15) | **<0.01** |
| Height (cm) | 159.6 (8.02) | 160.5 (8.04) | 160.9 (8.02) | 161.1 (7.97) | **<0.01** | 158.9 (7.69) | 160.3 (7.92) | 161.0 (8.03) | 161.9 (8.16) | **<0.01** |
| Weight (kg) | 60.7 (9.68) | 61.7 (9.81) | 62.2 (10.0) | 62.6 (10.14) | **<0.01** | 60.0 (9.40) | 61.5 (9.65) | 62.3 (9.97) | 63.4 (10.35) | **<0.01** |
| BMI (kg/m$^2$) | 23.8 (2.94) | 23.9 (2.89) | 24.0 (2.89) | 24.0 (2.94) | **<0.01** | 23.7 (2.94) | 23.9 (2.88) | 23.9 (22.89) | 24.1 (2.93) | **<0.01** |
| Albumin (g/dL) | 4.62 (0.28) | 4.63 (0.27) | 4.64 (0.27) | 4.65 (0.27) | **<0.01** | 4.6 (0.27) | 4.6 (0.27) | 4.6 (0.27) | 4.6 (0.27) | **<0.01** |
| Protein intake (g) | 36.8 (8.59) | 50.7 (8.42) | 62.8 (10.96) | 90.8 (34.25) | **<0.01** | 36.2 (8.08) | 50.2 (7.16) | 62.7 (9.29) | 92.0 (33.47) | **<0.01** |
| Creatinine (mg/dL) | 0.8 (0.24) | 0.8 (0.22) | 0.8 (0.20) | 0.8 (0.21) | **<0.01** | 0.8 (0.24) | 0.8 (0.19) | 0.8 (0.21) | 0.8 (0.22) | **<0.01** |
| Uric acid (mg/dL) | 4.7 (1.27) | 4.7 (1.28) | 4.7 (1.29) | 4.7 (1.28) | **<0.01** | 4.6 (1.25) | 4.7 (1.27) | 4.7 (1.29) | 4.8 (1.31) | **<0.01** |
| Total cholesterol (mg/dL) | 197.5 (35.5) | 197.6 (34.98) | 198.2 (35.45) | 198.2 (35.29) | **<0.01** | 197.7 (35.6) | 197.3 (35.07) | 197.8 (35.30) | 198.7 (35.24) | **<0.01** |

[1] Chronic kidney disease (CKD) was defined as the eGFR (estimated GFR) of less than 60 mL/min/1.73 m$^2$ on the basis of the National Kidney Foundation's Kidney Disease Outcome Quality Initiative working group definition (K/DOQI clinical practice guidelines for chronic kidney disease: evaluation, classification, and stratification. Am J Kidney Dis 2002;39(suppl 1):S1–266.)

[2] Patients with chronic kidney disease, cardiovascular disease and severe cancers (stomach cancer, liver cancer, colon cancer, lung cancer) are excluded from the study population. eGFR was calculated by MDRD formula [eGFR: 186×serum creatinine (mg/dL)$^{-1.154}$×age$^{-0.203}$×(0.742 if female)].

[3] Regular exercise was defined as performing regular exercise enough to sweat once a week or more.

[4] Passive smoking (among subjects who had never smoked) was determined by asking. "How many times do you indirectly inhale smoke from other people at home or your workplace?"

[5] Hypertension was defined as a person with anti-hypertensive medication or systolic blood pressure ≥ 140, diastolic blood pressure ≥ 90 mmHg, or the presence of history of hypertension.

[6] Diabetes was defined as fasting blood glucose ≥ 126 mg/ml or the presence of history of diabetes.

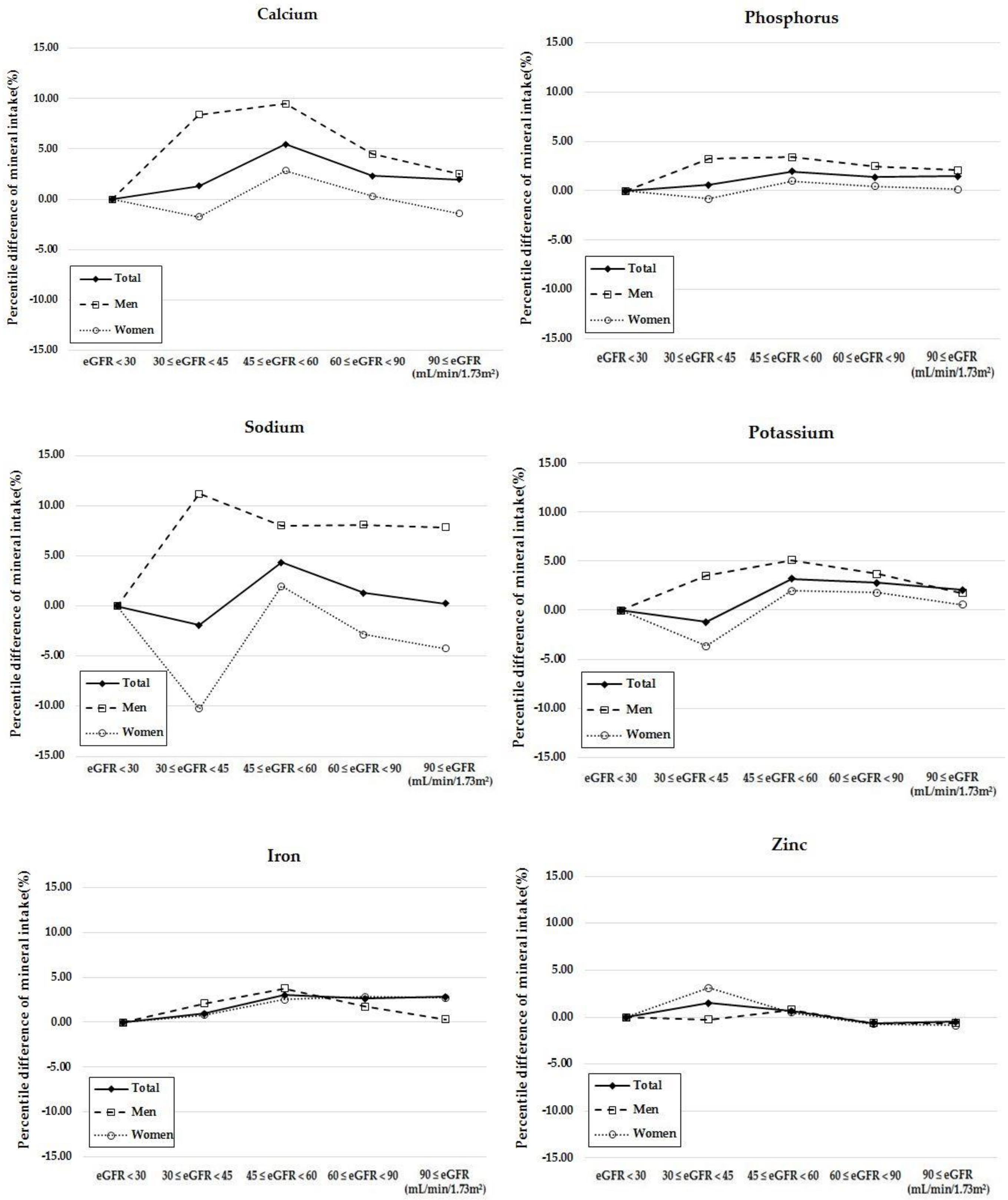


**Figure S1.** Percentile difference of geometric mean of dietary mineral intake according to CKD stages stratified by sex differences in the Health Examinees (HEXA) study of the Korea Genome and Epidemiologic Study (KoGES), 2005-2012

**<Reference>**


1. Levey, A.S.; Stevens, L.A.; Schmid, C.H.; Zhang, Y.L.; Castro, A.F.; Feldman, H.I.; Kusek, J.W.; Eggers, P.; Van Lente, F.; Greene, T. A new equation to estimate glomerular filtration rate. *Annals of internal medicine* **2009**, *150*, 604-612.
2. Stevens, L.A.; Claybon, M.A.; Schmid, C.H.; Chen, J.; Horio, M.; Imai, E.; Nelson, R.G.; Van Deventer, M.; Wang, H.-Y.; Zuo, L., *et al.* Evaluation of the ckd-epi equation in multiple races and ethnicities. *Kidney international* **2011**, *79*, 555-562.
